# Supplementary material for: Gender and other potential biases in peer review: cross-sectional analysis of 38 250 external peer review reports
Source: BMJ Open. 2020 Aug 20;10(8):e035058. doi: 10.1136/bmjopen-2019-035058 (PMC7440717; doi:10.1136/bmjopen-2019-035058)
Supplement: Supplementary data [file bmjopen-2019-035058supp001.pdf]

Supplementary materials

Table S1: P values from interaction tests of gender of the applicant with other variables, based on bivariable and multivariable models.

	Bivariable	Multivariable*
Gender of reviewer	0.011	0.037
Source of nomination of reviewer	0.71	0.17
Country of affiliation of reviewer	0.57	0.27
Age of applicant	0.74	0.67
Affiliation of the applicant	<0.001	0.003
Nationality of the applicant	0.51	0.92
Research topic	0.36	0.31
Change of guidelines	0.065	0.033

*Adjusted for all variables listed in Table 2 of the main paper.

Table S2: Definition of the field of research.
The field of research is based on the SNSF main discipline list.

Field of research	Main discipline
Medicine	Social medicine, Basic Medical Sciences, Experimental Medicine, Clinical Medicine, Preventive Medicine (Epidemiology/Early Diagnosis/Prevention), Social Medicine
Architecture	Art studies, musicology, theatre and film studies, architecture
Biology	Basic Biological Research, General Biology
Chemistry	Chemistry, Environmental Sciences
Economics	Economics, law
Engineering	Engineering Sciences
Geology	Earth Sciences
History	Theology and religious studies, history, classical studies, archaeology, prehistory and early history
Linguistics	Linguistics and literature, philosophy
Mathematics/Physics	Astronomy, Astrophysics and Space Sciences, Physics, Mathematics, Mathematics, Natural- and Engineering Sciences
Psychology	Psychology, educational studies
Sociology	Sociology, social work, political sciences, media and communication studies, health, Ethnology

Severin A, *et al. BMJ Open* 2020; 10:e035058. doi: 10.1136/bmjopen-2019-035058

Figure S1: Overall scores depending on research topic and gender of the applicant.

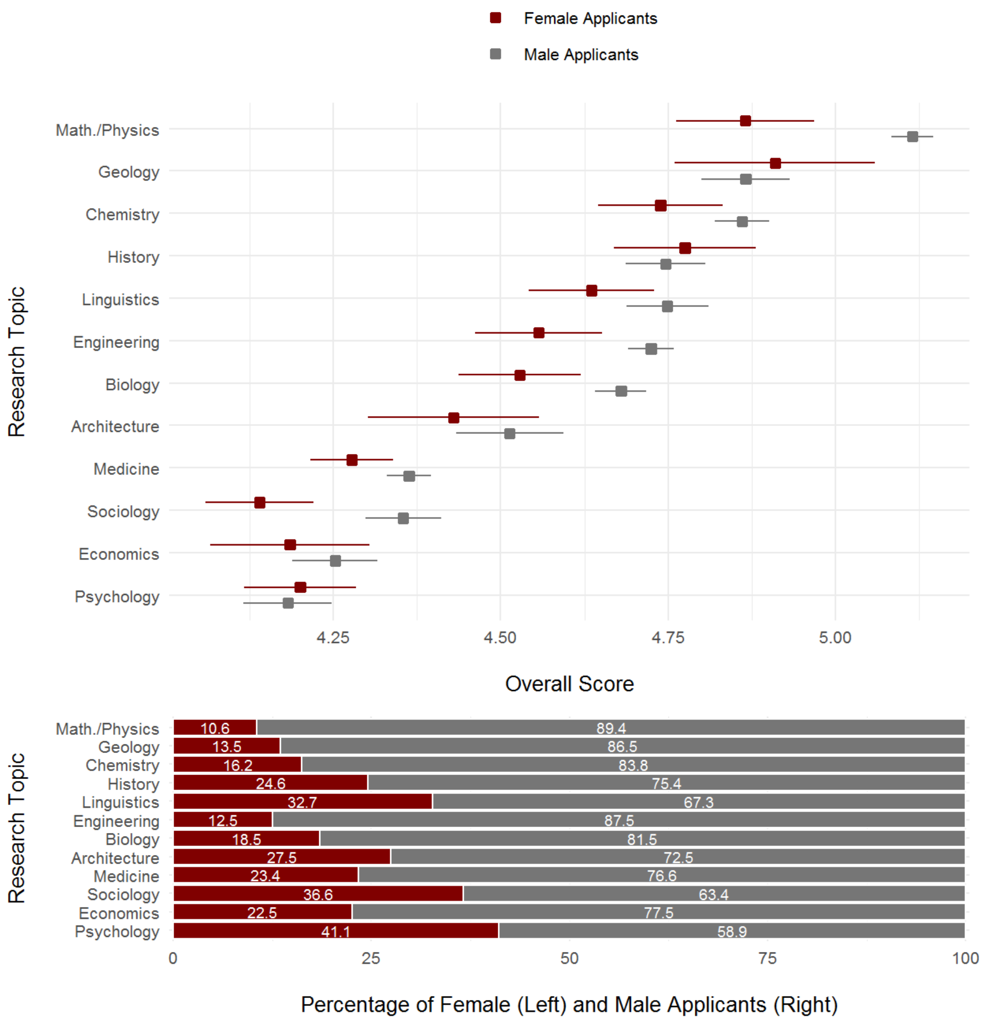

Upper panel: Average (mean) overall scores by research topic for female and male applicants; horizontal lines indicate Wald 95% confidence intervals. Lower panel: Gender distribution in the research topics.

Figure S2: Overall scores depending on the age and gender of the applicants.

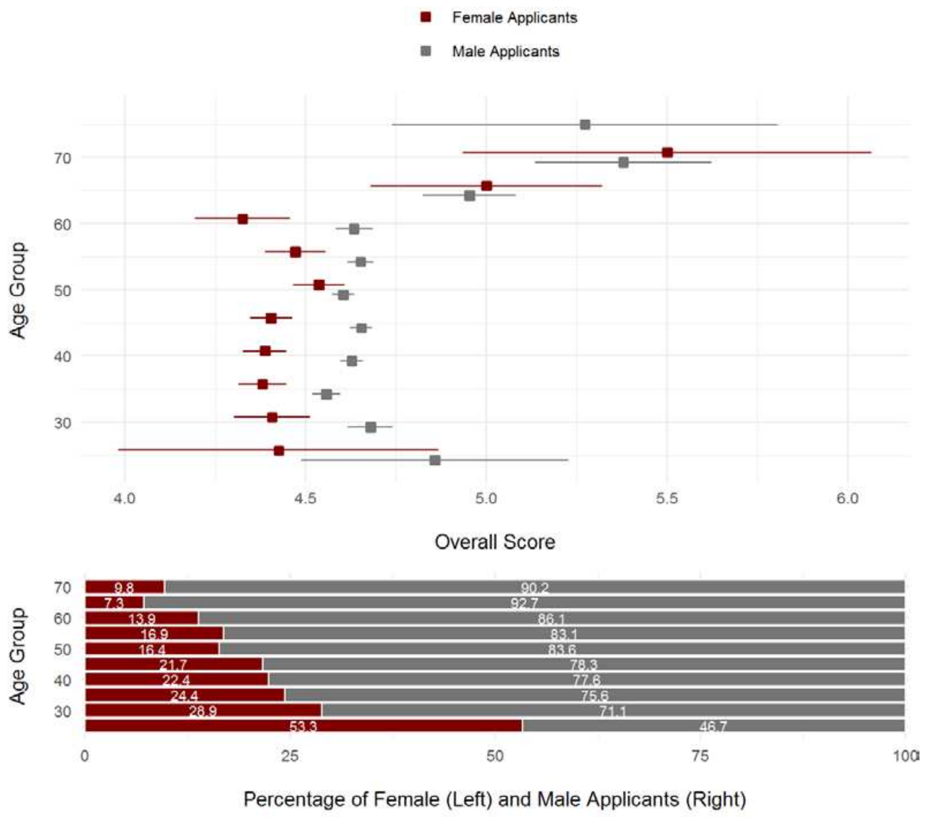

Upper panel: Average (mean) overall scores by five-year age group for female and male applicants; horizontal lines indicate Wald 95% confidence intervals. Lower panel: Gender distribution in the age groups.

Figure S3: Overall scores depending on the applicant's institution type and gender.

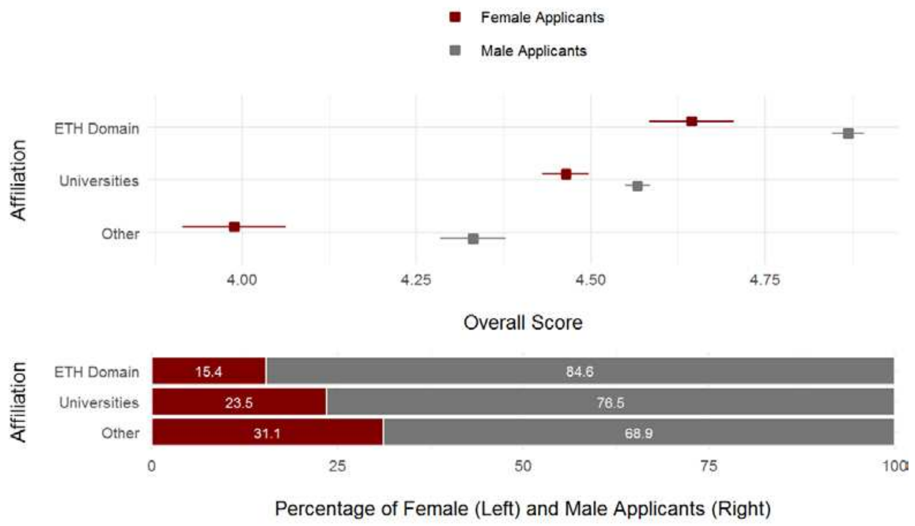

Upper panel: Average (mean) overall scores by affiliation times for female and male applicants; horizontal lines indicate Wald 95% confidence intervals. Lower panel: Gender distribution for the different affiliation types.

Figure S4: Average overall scores by nationality for female and male applicant; horizontal lines indicate Wald 95% confidence intervals.

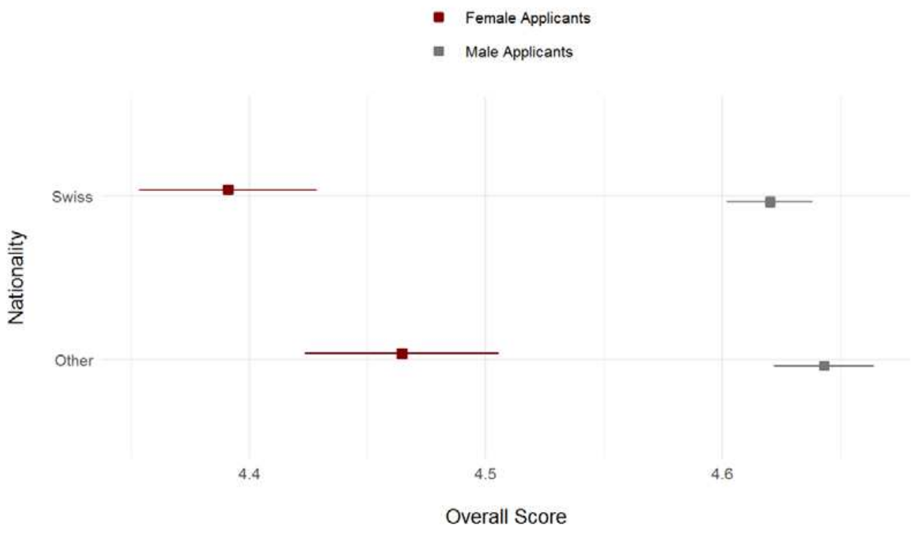

Figure S5: Average (mean) overall scores by application call deadline for female and male applicants; horizontal lines indicate Wald 95% confidence intervals.

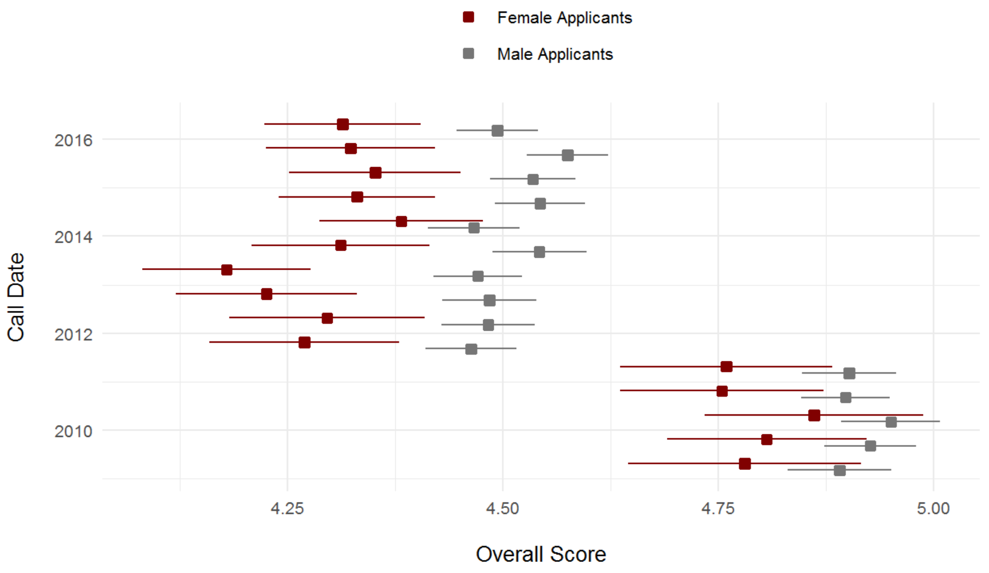

Severin A, *et al. BMJ Open* 2020; 10:e035058. doi: 10.1136/bmjopen-2019-035058

Text S1: Old and new evaluation forms. The new forms were implemented from the 1. October 2011 call onwards.

OLD FORM

1. Synopsis

	Excellent	Very Good	Good	Average	Satisfactory	Poor		Not considered
Current scientific interest and impact of the project								
Originality of the work								
Suitability and originality of the methods to be used								
Feasibility of the project								
Experience and past performance of the applicant								
Specific abilities of the applicants for the proposed project								

Overall assessment								

Comments regarding the overall assessment

2. Detailed evaluation

Current scientific interest and impact of the project

Originality of the work

Severin A, *et al. BMJ Open* 2020; 10:e035058. doi: 10.1136/bmjopen-2019-035058

Suitability and originality of the methods to be used

Feasibility of the project

Experience and past performance of the applicant

Specific abilities of the applicants for the proposed project

---

Other comments

NEW FORM

1. Synopsis

	outstanding	excellent	very good	good	average	poor		Not considered
Applicants' scientific track record and expertise								
Scientific relevance, originality and topicality								
Suitability of methods and feasibility								

Overall assessment								

Comments regarding the overall assessment

2. Detailed evaluation

Applicants' scientific track record and expertise

Scientific relevance, originality and topicality

Suitability of methods and feasibility

3. Further comments & declaration concerning conflicts of interests (will not be forwarded to applicants)

Confidential messages

The topic of the proposed project

Declaration concerning conflict of interests (comments, if applicable

---

Severin A, *et al. BMJ Open* 2020; 10:e035058. doi: 10.1136/bmjopen-2019-035058
