# Supplementary material for: Gender and other potential biases in peer review: cross-sectional analysis of 38 250 external peer review reports
Source: BMJ Open. 2020 Aug 20;10(8):e035058. doi: 10.1136/bmjopen-2019-035058 (PMC7440717; doi:10.1136/bmjopen-2019-035058)
Supplement: Author's manuscript [file bmjopen-2019-035058.draft_revisions.pdf]

BMJ Open

BMJ Open is committed to open peer review. As part of this commitment we make the peer review history of every article we publish publicly available.

When an article is published we post the peer reviewers' comments and the authors' responses online. We also post the versions of the paper that were used during peer review. These are the versions that the peer review comments apply to.

The versions of the paper that follow are the versions that were submitted during the peer review process. They are not the versions of record or the final published versions. They should not be cited or distributed as the published version of this manuscript.

BMJ Open is an open access journal and the full, final, typeset and author-corrected version of record of the manuscript is available on our site with no access controls, subscription charges or pay-per-view fees (http://bmjopen.bmj.com).

If you have any questions on BMJ Open's open peer review process please email info.bmjopen@bmj.com

BMJ Open

Gender and Other Potential Biases in Peer Review: Cross-sectional Analysis of 38,250 External Peer Review Reports

Journal:	*BMJ Open*
Manuscript ID	bmjopen-2019-035058
Article Type:	Original research
Date Submitted by the Author:	18-Oct-2019
Complete List of Authors:	Severin, Anna; University of Bern, ISPM<br>Martins, Joao; European Research Council<br>Heyard, Rachel; Swiss National Science Foundation<br>Delavy, François; Swiss National Science Foundation<br>Jorstad, Anne; Swiss National Science Foundation<br>Egger, Matthias; University of Bern, Institute of Social & Preventive Medicine
Keywords:	EPIDEMIOLOGY, STATISTICS & RESEARCH METHODS, Organisational development < HEALTH SERVICES ADMINISTRATION & MANAGEMENT

SCHOLARONE™
Manuscripts

I, the Submitting Author has the right to grant and does grant on behalf of all authors of the Work (as defined in the below author licence), an exclusive licence and/or a non-exclusive licence for contributions from authors who are: i) UK Crown employees; ii) where BMJ has agreed a CC-BY licence shall apply, and/or iii) in accordance with the terms applicable for US Federal Government officers or employees acting as part of their official duties; on a worldwide, perpetual, irrevocable, royalty-free basis to BMJ Publishing Group Ltd ("BMJ") its licensees and where the relevant Journal is co-owned by BMJ to the co-owners of the Journal, to publish the Work in this journal and any other BMJ products and to exploit all rights, as set out in our licence.

The Submitting Author accepts and understands that any supply made under these terms is made by BMJ to the Submitting Author unless you are acting as an employee on behalf of your employer or a postgraduate student of an affiliated institution which is paying any applicable article publishing charge ("APC") for Open Access articles. Where the Submitting Author wishes to make the Work available on an Open Access basis (and intends to pay the relevant APC), the terms of reuse of such Open Access shall be governed by a Creative Commons licence – details of these licences and which Creative Commons licence will apply to this Work are set out in our licence referred to above.

Other than as permitted in any relevant BMJ Author's Self Archiving Policies, I confirm this Work has not been accepted for publication elsewhere, is not being considered for publication elsewhere and does not duplicate material already published. I confirm all authors consent to publication of this Work and authorise the granting of this licence.

For peer review only

Gender and Other Potential Biases in Peer Review: Cross-sectional Analysis of 38,250 External Peer Review Reports

Anna Severin[1,2], João Martins[1,3], Rachel Heyard[1], François Delavy[1], Anne Jorstad[1], Matthias Egger[1,2]

[1]Swiss National Science Foundation, Bern, Switzerland

[2] Institute of Social and Preventive Medicine, University of Bern, Bern, Switzerland

[3] European Research Council Executive Agency, Brussels, Belgium

Corresponding Author:

Professor Matthias Egger MD MSc FFPH

Swiss National Science Foundation

Wildhainweg 3

3001 Bern, Switzerland

matthias.egger@ispm.unibe.ch

Abstract 274 words, main text 3913 words, strengths and limitations 126 words, 1 figure, 3 tables, 32 references, supplementary materials with 1 table, 5 figures and 1 text.

For peer review only - http://bmjopen.bmj.com/site/about/guidelines.xhtml

ABSTRACT

Objectives: To examine whether the gender of applicants and peer reviewers and other factors influence peer review of grant proposals submitted to a national funding agency.

Setting: Swiss National Science Foundation (SNSF).

Design: Cross-sectional analysis of peer review reports submitted 2009 to 2016 using linear mixed effects regression models adjusted for research topic, applicant's age, nationality, affiliation and calendar period.

Participants: External peer reviewers.

Primary outcome measure: Overall score on a scale from 1 (worst) to 6 (best).

Results: Analyses included 38,250 reports on 12,294 grant applications from medicine, Architecture, Biology, Chemistry, Economics, Engineering, Geology, History, Linguistics, Mathematics, Physics, Psychology and Sociology submitted by 26,829 unique peer reviewers. In univariable analysis, male applicants received more favourable evaluation scores than female applicants (+0.18 points; 95% CI 0.14-0.23), and male reviewers awarded higher scores than female reviewers (+0.11; 95% CI 0.08-0.15). Applicant-nominated reviewers awarded higher scores than reviewers nominated by the SNSF (+0.53; 95% CI 0.50-0.56), and reviewers from outside of Switzerland more favourable scores than reviewers affiliated with Swiss institutions (+0.53; 95% CI 0.49-0.56). In multivariable analysis, differences between male and female applicants were attenuated (to +0.08; 95% CI 0.04-0.13) whereas results changed little for source of nomination and affiliation of reviewers. The gender difference increased after September 2011, when new evaluation forms were introduced (P=0.033 from test of interaction).

Conclusions: Peer review of grant applications at SNSF might be prone to biases stemming from different applicant and reviewer characteristics. The SNSF abandoned the nomination of peer reviewers by applicants. The new form introduced in 2011 may inadvertently have given more emphasis to the applicant’s track record. We encourage other funders to conduct similar studies, in order to improve the evidence base for rational and fair research funding.

Keywords: peer review, bias, gender matching hypothesis, confounding, mixed effects models

For peer review only - http://bmjopen.bmj.com/site/about/guidelines.xhtml

For peer review only

STRENGTHS AND LIMITATIONS OF THIS STUDY

- This study was based on a large sample of peer review reports on project proposals from medicine and other disciplines submitted to the national Swiss funding agency.
- It is one of a few studies examining the interaction between gender of main applicant and gender of reviewers and the 'gender matching hypothesis', as well as the influence of other characteristics of applicants.
- This study only examined scores from peer review, but not the determinants of the final funding decision or the level of funding. It is therefore unclear whether the differences in scores analysed in the present study influenced funding decisions.
- This study was carried out by researchers affiliated with the funding agency and not by an independent group of researchers.

For peer review only

For peer review only - http://bmjopen.bmj.com/site/about/guidelines.xhtml

BACKGROUND

In public research funding, peer review of proposals by suitable experts is the accepted best practice for determining which projects are allocated funding. Peer review is an important element of quality assurance in the scientific community (1). Against this background, a wealth of literature is concerned with the question of the legitimacy of peer review decisions. Generally speaking, the legitimacy of funding decisions relies on a funder's ability to minimize bias in grant evaluations that results from factors that are unrelated to the actual quality of the grant applications (2).

Mandated by the Swiss Confederation, the Swiss National Science Foundation (SNSF) supports basic research and use-inspired basic research in all academic disciplines. The SNSF started monitoring its evaluation processes in 2006. The main funding scheme of the SNSF is project funding, which provides support to independent researchers who propose research on self-chosen topics (3). The proposals submitted to the SNSF are peer reviewed by at least two external experts.

Empirical studies suggest that the evaluation of applications is prone to biases that may relate to both applicant and reviewer characteristics (2)(4). Potential discrimination against women is the most frequently investigated bias in the context of grant peer review (5). In a natural experiment, a recent study of the Canadian Institutes of Health Research compared grant programmes with and without an explicit review of the track record of the principal investigator (6). The results showed that the gender gap in grant funding was due to less positive assessments of women as principal investigators, whereas the quality of the proposed research was similar for women and men (6). Of note, the SNSF introduced new evaluation forms and guidelines for peer reviewers in September 2011, which we describe in the Methods section.

The source of nomination of reviewers was also of interest: the foundation allowed grant applicants to suggest reviewers to evaluate submissions via a “positive list”. The names put forward on the list were then considered as potential reviewers, after a careful check for conflicts of interest (CoI). A study of the Australian Research Council found that applicant-nominated reviewers tended to give better ratings than panel-nominated reviewers (7). Finally, the SNSF frequently invites reviewers from abroad to review grant applications. An analysis of data from the Austrian Science Fund suggested that international peer reviewers

For peer review only - http://bmjopen.bmj.com/site/about/guidelines.xhtml

affiliated with research institutions located in countries known for high scientific productivity were generally more stringent than national reviewers (8).

To gain insights into gender and other potential biases in peer review, we analysed the database of the SNSF to examine the determinants of overall scores from external peer reviewers in project funding.

METHODS

Evaluation of Grant Applications at the SNSF

The evaluation consists of four steps (3). The administrative office first checks eligibility and assigns grant applications to two members of the National Research Council (referee and co-referee) based on their field of expertise. Second, eligible proposals are peer-reviewed by external experts. External reviewers were identified in several ways: (i) grant applicants suggested experts via a "positive list", (ii) the referee of the National Research Council suggested reviewers, (iii) the SNSF administrative offices proposed experts, and (iv) experts who declined to review suggested other reviewers (3). For each application, at least two external independent reviews were required.

The final choice of reviewers was made by the SNSF. Reviewers from the positive list were chosen only if they had the required expertise and there were no CoI. To exclude any CoI, SNSF employees checked whether reviewers had submitted an application for the same call, whether they had published with the applicants in the past five years and whether they work at the same institution or in a closely associated unit. Applicants could also submit a "negative list" of reviewers who, because of a possible CoI, should not be contacted. Providing a positive or a negative list was optional and the lists could include one or several names.

The peer review forms and assessment scale were changed in September 2011 to simplify the review, and to achieve a more equal distribution of scores, with fewer proposals in the top category. Up to September 2011, peer reviewers were asked to score six criteria: (1) current scientific interest and impact of the project; (2) originality of the work; (3) suitability of the methods; (4) work plan, feasibility, cost; (5) experience and past performance of the applicants; and (6) specific abilities of the investigators for the proposed project. Reviewers

For peer review only - http://bmjopen.bmj.com/site/about/guidelines.xhtml

were asked to "give a rating and provide explanatory comments" for each of the six criteria. In September 2011, new evaluation forms were introduced (3)(9), which asked experts to review proposals according to three criteria: 1) the applicants' scientific track-record and expertise; 2) the scientific relevance, originality and topicality of the proposed research and, in the case of use-inspired research, the research's broader impact; and 3) the suitability of the methods and feasibility. Furthermore, peer reviewers were asked to declare any CoI, and given the opportunity to submit confidential comments, which would not be seen by the applicants. Up to September 2011, reviewers scored each criterion and the proposal overall on a scale from 1 to 6: (1) poor, (2) satisfactory, (3) average, (4) good, (5) very good, and (6) excellent. In September 2011 the scale was changed to (1) poor, (2) average, (3) good, (4) very good, (5) excellent, and (6) outstanding. The two versions of the peer review form are reproduced in supplementary Text S1. Applications were not blinded and reviewers were therefore aware of applicant's gender and their track records.

In the third step of the evaluation, the two members of the Council (referee and co-referee) assessed the usefulness of the peer review reports and considered them when ranking the application relative to other proposals. In the fourth and final step, referee and co-referee presented their assessment at the meeting of the corresponding section of Council. Each application was then voted on and approved or rejected (3).

Data and Variables

We analysed the overall scores of external peer review reports submitted from 2009 to 2016. The outcome variable of interest was the overall evaluation score of a grant application given by external reviewers. The evaluation score ranged from 1 (worst) to 6 (best). Explanatory variables included meta-data on principle applicants and external peer reviewers, including source of reviewer (applicant-nominated vs. SNSF- nominated), gender of the applicant and gender of the reviewer (female vs. male) and country of affiliation of the reviewer (Switzerland vs. other). The mean ratio of female to male reviewers per grant application was 0.2. Eighteen percent of the grant applications had male-only external reviewers while only 1% had female-only external reviewers. SNSF-nominated experts included reviewers who were proposed by the referee, the SNSF office or by experts who declined to review. We also considered the research topic of a grant application, type of

For peer review only

institutional affiliation and age of the applicant. Finally, we introduced a dummy variable to group applications submitted before and after September 2011.

Statistical Analysis

We used a linear mixed effects model to examine the effect of explanatory variables on the overall peer-review scores (10). This model was chosen because the data are clustered and hierarchical (11). Grant applications received two or more independent reviews, some reviewers had reviewed more than one application and many applicants had submitted more than one grant application over the study period, causing evaluation scores to be clustered at the levels of research projects, reviewers and applicants. We therefore introduced random intercepts for the identifiers of the reviewer, the applicant and the project in the model, thus taking into account the dependence between clustered scores (12).

We ran crude and adjusted models. The latter were adjusted for gender of the applicant and reviewer, source of reviewers, country of affiliation of the reviewer, the applicant's age (per 10 year increase), affiliation (Swiss Federal Institutes of Technology and associated institutions [ETH domain], Cantonal university, other) and nationality (Swiss vs. other), the field of research (12 categories), and the period of submission of the proposal (before or after the change in peer review forms and scale). To make adjusted and crude estimates comparable, we performed a complete case analysis by deleting peer review reports with missing values for any of the relevant variables. The complete case data included 37,979 reviews (99.3%). In further analyses, we examined interactions between the gender of the applicant and the gender of the reviewer, and other variables, by including interaction terms in the linear mixed models. We thus examined the 'gender matching hypothesis', which stipulates that female peer reviewers give higher scores to female researchers and that male reviewers do the same for male applicants (13). We used likelihood ratio tests to assess the strength of the evidence for interactions.

We present crude and adjusted regression coefficients, which reflect differences in peer review scores with their 95% confidence interval (CI). The notebook of the analysis, including a summary of the different statistical models, is available online at www.git.io/fhaJx.

For peer review only - http://bmjopen.bmj.com/site/about/guidelines.xhtml

Patient and Public Involvement

This analysis was based on peer review reports submitted to a national research funder. No patients were involved in developing the research question, outcome measures and overall design of the study. Due to the anonymous nature of the data, we were unable to disseminate the results of the research directly to study participants.

RESULTS

We analysed the summary scores of 38,250 external peer review reports on 12,294 project grant applications across all disciplines that were submitted 2006 to 2016 by 26,829 unique reviewers from Switzerland and abroad. The average number of reviews per grant application was 3.1, applicants submitted an average of 2.1 grant applications and reviewers reviewed an average of 1.4 applications. The complete case mixed effects regression analyses were based on 37,989 reviews (99.3%).

Applicant characteristics

The 12,294 proposals were submitted by 5,820 applicants: 4,514 (77.6%) men and 1,306 (22.4%) women (Table 1). Most applicants were based at Cantonal universities, were Swiss, and the largest number was from medicine. Female applicants were younger than men and more likely to be affiliated with other institutions (for example universities of applied sciences, the arts or teacher education) than with the Federal ETH domain or the Cantonal universities. Women were also more likely to work in medicine, the social sciences and humanities (psychology, sociology, linguistics) than in STEM disciplines (Science, Technology, Engineering, and Mathematics) or biology (Table 1).

Peer review scores across groups of applicants and reviewers

Distributions of overall peer review scores were somewhat skewed, with applications more frequently being awarded high evaluation scores than low scores (see notebook at www.git.io/fhaJx). Male principle applicants received higher evaluation scores than female principle applicants (Table 2). Similarly, the analysis of evaluation scores by gender of the reviewer showed that male reviewers tended to award higher scores than female reviewers. Applicant-nominated reviewers awarded higher scores than SNSF-nominated reviewers, and

For peer review only

For peer review only - http://bmjopen.bmj.com/site/about/guidelines.xhtml

reviewers affiliated with institutions outside Switzerland awarded higher evaluation scores than reviewers affiliated with Swiss institutions.

To further explore gender differences in peer review scores, we stratified analyses of peer review reports by research field, applicant age and applicant affiliation. There were important differences in evaluation scores across research fields. Grant applications in the natural and technical sciences or in linguistics and history received higher evaluation scores than applications from medicine, sociology or psychology (supplementary Figure S1). Gender differences in scores were more pronounced for some research topics (for example mathematics and physics and engineering, biology and medicine, sociology) than others (for example geology, history, psychology). Female applicants were underrepresented (below 50 percent) in all research topics (lower panel of supplementary Figure S1).

Applicants aged 60 years or older received the highest evaluation scores, independent of their gender. For the younger age groups, female applicants consistently received lower evaluation scores than male applicants (supplementary Figure S2). Female applicants were under represented across all age groups, except for the youngest age group, and representation was particularly low in older age groups (lower panel of supplementary Figure S2). Applications submitted by applicants affiliated with the ETH Domain received higher evaluation scores than applications from Cantonal universities or from other research institutions. Gender differences in scores were evident for all three affiliations, and women were under represented for all affiliations (supplementary Figure S3).

Analysis of the nationality of the applicant showed that grant applications submitted by Swiss applicants received slightly lower scores than those submitted by applicants with other nationalities, with a similar gap between genders (supplementary Figure S4). Finally, supplementary Figure S5 shows that applications submitted before the new forms were introduced received higher scores than applications evaluated later.

Linear Mixed-Effects Models

Table 3 shows crude and adjusted differences in peer review scores by characteristics of applicants, reviewers and research proposals. In the crude model, the difference between male and female applicants was 0.18 points favouring men. More substantial differences of 0.53 points were observed for source of reviewer (0.53 points higher if the reviewer was nominated by the applicants) and country of affiliation of the reviewer (0.53 higher for

For peer review only - http://bmjopen.bmj.com/site/about/guidelines.xhtml

reviewers from outside Switzerland). Substantial differences were also observed across disciplines. For example, scores were on average 0.68 points higher in mathematics and physics than in medicine, but 0.12 point lower in psychology than in medicine (Table 3). Compared to crude differences, most adjusted differences were smaller. For example, the adjusted difference between male and female applicants was reduced from 0.18 to 0.08 points. One exception was the difference observed between proposals evaluated before or after the introduction of the peer review forms in September 2011 (0.43 points higher scores before the introduction in both analyses).

Interactions between gender of the applicants and other variables

We examined possible interactions between the genders of the applicants with the other fixed-effect variables in the model shown in Table 2. In other words, we examined whether the differences observed between female and male applicants varied across the levels of the other variables. We found that male reviewers gave higher scores both to male and female applicants than female reviewers, but this difference was considerably greater for male than for female applicants. Figure 1 shows the predicted values of the overall score from the bivariable model (P=0.011 from test of interaction). There was some evidence that the gender difference in scores became larger after the introduction of the new evaluation form (P=0.065, Figure 1). There was also strong evidence for an interaction (P<0.0001) between gender of the first applicant and his or her affiliation: the gender differences in scores were smallest for applicants based at one of the Cantonal universities, larger for the ETH domain and most pronounced for other institutions of higher education (for example universities of applied sciences, the arts or teacher education, see Figure 1). The interaction P values from the adjusted models were 0.037 (gender of peer reviewer), 0.003 (affiliation of applicant) and 0.033 (change of evaluation form). All P values from the bivariable and multivariable interaction tests are shown in supplementary Table S1.

For peer review only - http://bmjopen.bmj.com/site/about/guidelines.xhtml

DISCUSSION

This study of 38,250 distinct grant reviews on 12,294 different applications across all disciplines, which were submitted to the SNSF between 2009 and 2016 by 5,832 unique applicants is to the best of our knowledge one of the largest studies of peer review reports on research proposals ever conducted.

We show that female applicants received lower scores than male applicants. The gender difference was attenuated in multivariable analysis: it was partly explained by the fact that women were under represented among applicants in the fields and institutions whose proposals were rated highly, for example mathematics and physics, and institutions of the ETH domain. Our finding is in line with the meta-analysis of 21 studies by Bornmann et al., who found small but robust gender differences in grant award procedures with men globally having greater odds of receiving grants than women (30). Of note, applying a different statistical method, Marsh et al. found no gender differences for most effect sizes from Bornmann et al. (31). Kaatz et al. using data from the National Institutes of Health (NIH) did a text analysis of critiques for funded and unfunded NIH grant applications at the University of Wisconsin-Madison and found that reviewers assigned significantly worse scores for research approach, significance and priority to female than male applicants. The authors concluded that reviewers implicitly hold male and female applicants to different standards of evaluation (32).

Although a statistically substantial proportion of the gender gap in our study was explained by other factors, these factors might be a reflection of the leaky pipeline, i.e. “the phenomenon of women dropping out of research and academic careers at a faster rate than men” (14), which is well documented for Switzerland (15,16). The academic pipeline in Switzerland is particularly leaky in the life sciences, social sciences and humanities. In STEM the rate of dropout of women is less pronounced, but they are a minority from the start: among PhD students only about 20% are women, whereas in the social sciences, humanities, and the life sciences the majority of doctoral students are women (16).

Ceci and Williams (17) have argued that several factors are responsible for the under representation of women, including fertility choices and work-home balance issues, which affect women in all fields, not just STEM, whereas other factors such as career preferences and gender differences in mathematics achievement and attitudes impact particularly

For peer review only - http://bmjopen.bmj.com/site/about/guidelines.xhtml

women in math-based fields. The latter may in turn be influenced by cultural stereotypes and gender roles that lead to socialization processes that shape performance (18,19). In Switzerland, men are assigned the role of 'main breadwinner', resulting in uneven distribution of housework, unfavourable fiscal policies for households with two earners, and a lack of affordable child care (15,20). At the same time, the post-doc “bubble” in Switzerland is taking place in a situation of full employment and relative shortage of skilled labour (21).

A noteworthy finding of our study was the interaction between the gender of applicants and peer reviewers. In contrast to Jayasinghe and colleagues (13), who analysed 7,153 reviewer ratings at the Australian Research Council large grant programme and other smaller studies (22)(23), we found evidence supporting the ‘gender matching hypothesis’. Male reviewers gave systematically higher ratings to male applicants than to female applicants, whereas the same phenomenon could not be observed for female reviewers. One possible explanation is that, if such matching bias was present, male reviewers will have favoured male applicants, despite the fact that the proposals from male and female applicants might have been of similar quality. Alternatively, assuming proposals from male applicants were in fact stronger, female reviewers could have been biased against men and downgraded their proposals.

Male reviewers may have given more weight to the track record of applicants than female reviewers. In this context, it is interesting that the gender gap became wider after September 2011, when new evaluation forms for external peer review were introduced. The new guidelines and form separated the criteria related to the applicants, and the criteria related to the proposed project. On the new form, the applicant’s track record was the first criterion out of a total of three, whereas it was the fifth out of six criteria on the old form. Although this was not intended, the reform may have led to reviewers giving more weight to the track record of applicants, due to its prominence on the new form. Based on a Canadian Institutes of Health Research study, which showed that the gender gap in grant funding was due to less positive assessments of women as principal investigators whereas the quality of the proposed research was similar for women and men (6), Raymond and Goodman asked funders to “evaluate projects, not people” (24). We are planning additional analyses to examine whether at the SNSF the same phenomenon is at play, i.e. whether the gender gap is driven by the assessments of the track record. Furthermore, the SNSF is discussing changes to the peer review form.

For peer review only - http://bmjopen.bmj.com/site/about/guidelines.xhtml

Our results confirm those from the Australian Research Council, which showed that applicant-nominated reviewers tended to give substantially higher ratings than panel-nominated reviewers (7). A study of peer review in biomedical journals also found that author-nominated reviewers submitted more favourable recommendations than editor-nominated reviewers (25). This difference may be interpreted in several ways. First, nominated reviewers may have a CoI that remained undetected in the SNSF CoI examination. Alternatively, applicants may nominate reviewers who are more familiar with their field than reviewers nominated by the SNSF, and thus more able to recognize the impact and importance of the proposed research. Like the Australian Research Council, the SNSF felt that bias was the most likely explanation and decided to discontinue the use of the "positive list" in 2016. Of note, applicants can still submit a "negative list" of reviewers that should not be used because of perceived CoI.

Peer reviewers affiliated with a Swiss research institution gave lower scores than reviewers from outside Switzerland. A study of the Austrian Science Fund suggested that reviewers from countries with high scientific productivity were more stringent than national reviewers (8). Switzerland belongs to the most productive countries in terms of research output (26) and this might explain why reviewers affiliated with Swiss research institutions award lower evaluation scores than reviewers from abroad. In contrast to the Austrian Science Fund study (8), the Australian data showed that reviewers affiliated with an institution in the United States of America (USA) were more lenient than reviewers affiliated with institutions located in the United Kingdom, Germany or Australia (27), despite the fact that the USA is the country with the highest research output globally (26). Other explanations for the lower scores awarded by Swiss reviewers include greater knowledge of the local research capacity and expertise, or bias, if reviewers based in Switzerland downgraded the proposals of their competitors.

Our study has several limitations. First and most importantly, we did not examine the determinants of the final funding decision or the level of funding. It is therefore unclear whether the differences in scores analysed in the present study influenced funding decisions. Such analyses are planned for the future. Second, this is an observational study and it is therefore difficult to infer causality from the associations observed. Chance, bias, and confounding variables must be considered as possible explanations for associations between reviewer and applicant characteristics and overall scores (28). We tried to control

For peer review only - http://bmjopen.bmj.com/site/about/guidelines.xhtml

the influence of confounding variables by adjusting for these in regression models. We are considering randomized experiments to test certain interventions (e.g., blinding) in order to prevent or reduce gender effects for the future. Third, our results are relevant to the Swiss context, but may not be applicable to other countries. Fourth, we did not attempt to rate the expertise of the reviewers, and adjust for the differences in individual reviewers scores based on their previous performance. Fifth, this study was carried out by researchers affiliated with the SNSF and not by an independent research institution. As studies might be influenced by the expectations of the researchers of the study, the credibility of the results might be reduced. We address this by making the data available for replication. Finally, we examined project funding only, but not career funding or programme funding.

CONCLUSIONS

In conclusion, our results had important implications for the evaluation of project grant proposals: we abandoned the nomination of peer reviewers by applicants, and make members of evaluation panels aware of the other factors, including the gender and affiliation of reviewers, that can influence review scores. We encourage all funding bodies to contribute to research on potential biases in research funding, and ways of preventing them (29).

For peer review only

FOOTNOTES

Acknowledgements: Earlier results from this analysis were presented at the 5th International Congress on Peer Review and Scientific Publication, Chicago, Illinois, USA; September 10-12, 2017. We are grateful to Angelika Kalt, Benjamin Rindlisbacher and Barbara Curdy-Korrodi for helpful comments on previous versions of this paper, and to Andreas Limacher and Lukas Bütikofer (Clinical Trials Unit of the Faculty of Medicine of the University of Bern) for advice on the statistical analyses.

Contributors: AS, JM and ME conceived the study. JM and RH performed statistical analyses. FD and AJ contributed to data management and statistical analyses. AS and JM wrote the first draft of the paper, which was revised by ME, AS and RH. All authors contributed to and approved the final version.

Funding: This work was supported by the SNSF (internal funds and grant number 174281).

Competing interests: The authors are employees of the SNSF; they declare that they have no other competing interests.

Patient consent: Not applicable.

Ethics approval: Under Swiss law, not ethics approval is required for this type of study. Peer reviewers did not provide consent. No peer reviewer, applicant or proposal can be identified from this report.

Data sharing statement: The data analysed in this study are available to others on request for an approved research project, after signing a data sharing agreement.

For peer review only

References

1. Harman G. The Management of Quality Assurance: A Review of International Practice. Higher Education Quarterly 52(4):345 - 364. 2002;52(4):345–64.

2. Bornmann L, Daniel H-D. Gatekeepers of science - Effects of external reviewers' attributes on the assessments of fellowship applications. J Informetr. 2007 Jan;1(1):83–91.

3. Swiss National Science Foundation. Funding Regulations. Regulations of the Swiss National Science Foundation on research grants. Version 1.1.2016 [Internet]. 2016. Available from: http://www.snf.ch/SiteCollectionDocuments/allg_reglement_16_e.pdf

4. Demicheli V, C DP. Peer review for improving the quality of grant applications (Review). 2008;(2).

5. Mutz R, Bornmann L, Daniel H-D. Does Gender Matter in Grant Peer Review? An Empirical Investigation Using the Example of the Austrian Science Fund. Z Psychol-J Psychol. 2012;220(2):121–9.

6. Witteman HO, Hendricks M, Straus S, Tannenbaum C. Are gender gaps due to evaluations of the applicant or the science? A natural experiment at a national funding agency. The Lancet. 2019 Feb;393(10171):531–40.

7. Marsh HW, Bonds NW, Jayasinghe UW. Peer review process: Assessments by applicant-nominated referees are biased, inflated, unreliable and invalid. Aust Psychol. 2007 Mar;42(1):33–8.

8. Fischer C, Reckling FJ. Factors Influencing Approval Probability in Austrian Science Fund (FWF) Decision-Making Procedures - FWF Stand-Alone Projects Programme, 1999 to 2008 [Internet]. Rochester, NY: Social Science Research Network; 2010 Dec [cited 2019 May 5]. Report No.: ID 1725985. Available from: https://papers.ssrn.com/abstract=1725985

9. Project funding - SNF [Internet]. [cited 2019 May 25]. Available from: http://www.snf.ch/en/theSNSF/evaluation-procedures/project-funding/Pages/default.aspx

10. Bates D, Maechler M, Bolker BM, Walker SC. Fitting Linear Mixed-Effects Models Using lme4. J Stat Softw. 2015 Oct;67(1):1–48.

11. Jayasinghe UW, Marsh HW, Bond N. A multilevel cross-classified modelling approach to peer review of grant proposals: the effects of assessor and researcher attributes on assessor ratings. Journal of the Royal Statistical Society: Series A (Statistics in Society). 2003 Oktober;166(3):279–300.

12. Harrison XA, Donaldson L, Correa-Cano ME, Evans J, Fisher DN, Goodwin CED, et al. A brief introduction to mixed effects modelling and multi-model inference in ecology. PeerJ. 2018;6:e4794.

13. Jayasinghe UW, Marsh HW, Bond N. A multilevel cross-classified modelling approach to peer review of grant proposals: the effects of assessor and researcher attributes on assessor ratings. J Royal Statistical Soc A. 2003 Oct;166(3):279–300.

14. Directorate-General for Research and Innovation. She Figures 2015 [Internet]. 2015. 224 p. Available from: https://ec.europa.eu/research/swafs/index.cfm?pg=library&lib=gender_equality

For peer review only

For peer review only - http://bmjopen.bmj.com/site/about/guidelines.xhtml

15. Bataille P, Le Feuvre N, Morales SK. Should I stay or should I go? The effects of precariousness on the gendered career aspirations of postdocs in Switzerland. Eur Educ Res J. 2017 May;16(2–3):313–31.

16. Schubert F, Engelage S. Wie undicht ist die Pipeline? Wissenschaftskarrieren von promovierten Frauen. Köln Z Soziol. 2011 Sep;63(3):431–57.

17. Ceci SJ, Williams WM. Understanding current causes of women's underrepresentation in science. Proc Natl Acad Sci U S A. 2011 Feb 22;108(8):3157–62.

18. Else-Quest NM, Hyde JS, Linn MC. Cross-national patterns of gender differences in mathematics: A meta-analysis. Psychological Bulletin. 2010 Jan;136(1):103–27.

19. Eagly AH, Steffen VJ. Gender Stereotypes Stem From the Distribution of Women and Men Into Social Roles. :20.

20. Domestic activities (time used in) - UNECE Statistical Glossary - UNECE Statswiki [Internet]. [cited 2019 May 12]. Available from: https://statswiki.unece.org/pages/viewpage.action?pageId=92211215

21. Wanner P, Zufferey J, Fioretta J. The impact of migratory flows on the Swiss labour market. A comparison between in- and outflows. Migr Lett. 2016 Sep;13(3):411–26.

22. Bornmann L, Daniel H-D. Gatekeepers of science—Effects of external reviewers' attributes on the assessments of fellowship applications. Journal of Informetrics. 2007 Jan;1(1):83–91.

23. Sonnert G. What Makes a Good Scientist?: Determinants of Peer Evaluation among Biologists. Social Studies of Science. 1995;25(1):35–55.

24. Raymond JL, Goodman MB. Funders should evaluate projects, not people. The Lancet. 2019 Feb;393(10171):494–5.

25. Schroter S, Tite L, Hutchings A, Black N. Differences in review quality and recommendations for publication between peer reviewers suggested by authors or by editors. JAMA-J Am Med Assoc. 2006 Jan 18;295(3):314–7.

26. Country outputs | Nature Index [Internet]. [cited 2019 May 14]. Available from: https://www.natureindex.com/country-outputs

27. Wood F. The peer review process [Internet]. Canberra: Australian Govt. Pub. Service; 1997 [cited 2019 May 5] p. 189. Available from: https://trove.nla.gov.au/version/45600880

28. Smith GD, Ebrahim S. Data dredging, bias, or confounding - They can all get you into the BMJ and the Friday papers. Br Med J. 2002 Dec 21;325(7378):1437-+.

29. Tricco AC, Thomas SM, Antony J, Rios P, Robson R, Pattani R, et al. Strategies to Prevent or Reduce Gender Bias in Peer Review of Research Grants: A Rapid Scoping Review. PLoS One. 2017 Jan 6;12(1):e0169718.

30. Bornmann L, & Mutz R, & Daniel HD, Gender differences in grant peer review: A meta-analysis. Journal of Informetrics 2007;1(3):226–238.

31. Marsh HW, Bornmann L, Mutz R, Daniel HD & O'Mara A. Gender Effects in the Peer Reviews of Grant Proposals: A Comprehensive Meta-Analysis Comparing Traditional and Multilevel

For peer review only

Approaches. Review of Educational Research 2009;79(3):1290-1326. doi:10.3102/0034654309334143.

32. Kaatz A, Lee Y-G, Potvien A, Magua W, Filut A, Bhattachary A, Leatherberry RX, DCarnes M. Analysis of NIH R01 Application Critiques, Impact and Criteria Scores: Does the Sex of the Principal Investigator Make a Difference? Academic Medicine 2016;91(8):1080-1088.

For peer review only

Legend to Figure

Figure 1: Gender differences in external evaluation scores by gender of the expert reviewer, affiliation and period of submission of the proposal.

Predicted values from bivariable (unadjusted) model are shown, together with their 95% confidence intervals. Scores range from 1 (worst) to 6 (best). Average (mean) overall scores are shown, horizontal lines indicate Wald 95% confidence intervals.

For peer review only

For peer review only - http://bmjopen.bmj.com/site/about/guidelines.xhtml

Table 1. Characteristics of applicants who submitted grant applications to the Swiss National Science Foundation between 2009 and 2016, stratified by gender.

	Male applicants (n = 4,514, 78%)	Female applicants (n = 1,306, 22%)
Age (mean, SD)	48.24 (8.63)	46.23 (8.27)
Affiliation		
ETH Domain	1,195 (85%)	219 (15%)
Other	481 (68%)	224 (32%)
Universities (reference)	2,838 (77%)	863 (23%)
Nationality		
Other than Swiss	1,896 (77%)	573 (23%)
Swiss	2,618 (78%)	733 (22%)
Field of research		
Medicine (reference)	1,029 (76%)	317 (24%)
Architecture	146 (72%)	56 (28%)
Biology	611 (83%)	129 (17%)
Chemistry	378 (83%)	76 (17%)
Economics	290 (78%)	84 (22%)
Engineering	527 (88%)	74 (12%)
Geology	144 (86%)	24 (14%)
History	209 (75%)	68 (25%)
Linguistics	203 (67%)	102 (33%)
Mathematics / Physics	491 (90%)	56 (10%)
Psychology	223 (58%)	164 (42%)
Sociology	263 (63%)	156 (37%)

The characteristics refer to the first submission of a project grant proposal during the study period. Numbers (%) are shown unless otherwise indicated. Analysis based on 5,820 unique applicants without missing values.

only

For peer review only - http://bmjopen.bmj.com/site/about/guidelines.xhtml

Table 2: Mean of overall score by groups of applicants and peer reviewers.

Group	No. of peer review reports	Mean overall score (SD)
Female Applicants	7,764	4.42 (1.25)
Male Applicants	30,455	4.63 (1.22)
Female Reviewers	7,591	4.44 (1.26)
Male Reviewers	30,659	4.63 (1.22)
Applicant-nominated Reviewers	8,755	5.12 (1.00)
SNSF-nominated Reviewers	29,495	4.43 (1.25)
International-based Reviewers	29,423	4.71 (1.19)
National-based Reviewers	8,604	4.16 (1.28)

SD, standard deviation.

Results based on 38,250 peer review reports.

For peer review only

Table 3: Crude and adjusted differences in external peer review evaluation scores by characteristics of applicants, reviewers and research proposals.

Variable	Number of reviews analysed	Unadjusted difference (95% CI)	P	Adjusted difference (95% CI)	P
Gender of the applicant			<0.001		<0.001
Male	30,263	0.18 (0.14 – 0.23)		0.08 (0.04 – 0.13)	
Female	7,716	0		0	
Gender of the reviewer			<0.001		<0.001
Male	30,442	0.11 (0.08 – 0.15)		0.08 (0.05 – 0.11)	
Female	7,537	0		0	
Source of nomination of reviewer			<0.001		<0.001
Applicant	8,688	0.53 (0.50 – 0.56)		0.49 (0.46 – 0.51)	
Office	29,291	0		0	
Country of affiliation of reviewer			<0.001		<0.001
Outside Switzerland	29,384	0.53 (0.49 – 0.56)		0.47 (0.44 – 0.50)	
Switzerland	8,595	0		0	
Age of the applicant	37,989		<0.001		<0.001
Per 10 year increase		0.06 (0.03 – 0.08)		0.05 (0.03 – 0.07)	
Affiliation of the applicant			<0.001		<0.001
ETH Domain	9,960	0.30 (0.26 – 0.34)		0.11 (0.07 – 0.16)	
Other	4,075	-0.24 (-0.30 - -0.19)		-0.19 (-0.25 – -0.14)	
Universities	23,944	0		0	
Nationality of the applicant			0.155		0.143
Other than Swiss	16,545	0.03 (-0.01 – 0.06)		-0.03 (-0.06 – 0.01)	
Swiss	21,434	0		0	
Field of research			<0.001		<0.001
Medicine	7,540	0		0	
Architecture	1,391	0.13 (0.03 – 0.24)		0.15 (0.05 – 0.25)	
Biology	3,872	0.30 (0.24 – 0.36)		0.27 (0.21 – 0.33)	
Chemistry	3,244	0.46 (0.39 – 0.53)		0.24 (0.17 – 0.31)	
Economics	2,171	-0.09 (-0.17 – -0.01)		-0.01 (-0.09 – 0.06)	
Engineering	4,880	0.32 (0.25 – 0.38)		0.07 (0.00 – 0.13)	
Geology	1,167	0.50 (0.39 – 0.60)		0.25 (0.14 – 0.35)	
History	2,053	0.35 (0.27 – 0.44)		0.32 (0.24 – 0.40)	
Linguistics	2,244	0.30 (0.22 – 0.38)		0.26 (0.18 – 0.34)	
Mathematics / Physics	3,979	0.68 (0.62 – 0.75)		0.45 (0.39 – 0.52)	
Psychology	2,458	-0.12 (-0.20 – -0.05)		-0.08 (-0.15 – 0.00)	
Sociology	2,980	-0.06 (-0.13 – 0.02)		0.01(-0.06 – 0.08)	
Introduction of reviewer guidelines			<0.001		<0.001
Before introduction	11,151	0.44 (0.41 – 0.47)		0.43 (0.40 – 0.46)	
After introduction	26,828	0		0	

95% CI, 95% confidence interval. Results from linear mixed effects models based on 37,979 complete peer review reports.

For peer review only - http://bmjopen.bmj.com/site/about/guidelines.xhtml

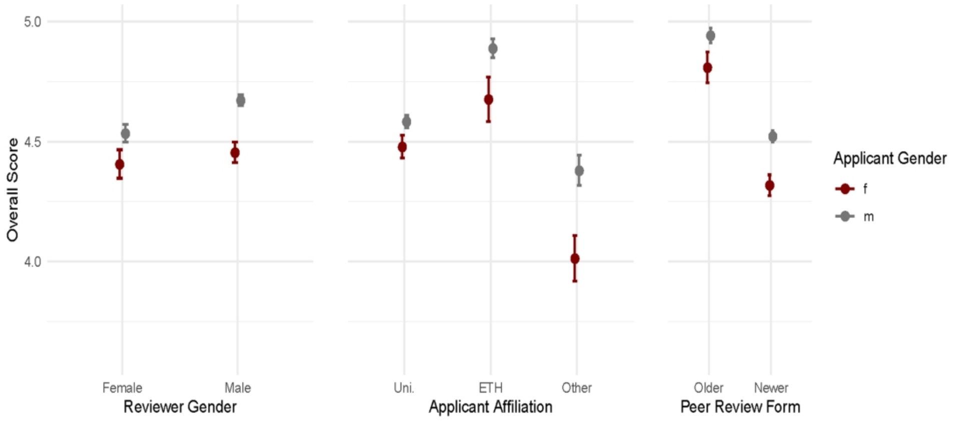

Figure 1: Gender differences in external evaluation scores by gender of the expert reviewer, affiliation and period of submission of the proposal.
Predicted values from bivariable (unadjusted) model are shown, together with their 95% confidence intervals. Scores range from 1 (worst) to 6 (best). Average (mean) overall scores are shown, horizontal lines indicate Wald 95% confidence intervals.

Supplementary materials

Table S1: P values from interaction tests of gender of the applicant with other variables, based on bivariable and multivariable models.

	Bivariable	Multivariable*
Gender of reviewer	0.011	0.037
Source of nomination of reviewer	0.71	0.17
Country of affiliation of reviewer	0.57	0.27
Age of applicant	0.74	0.67
Affiliation of the applicant	<0.001	0.003
Nationality of the applicant	0.51	0.92
Research topic	0.36	0.31
Change of guidelines	0.065	0.033

*Adjusted for all variables listed in Table 2 of the main paper.

peer review only

For peer review only - http://bmjopen.bmj.com/site/about/guidelines.xhtml

Figure S1: Overall scores depending on research topic and gender of the applicant.

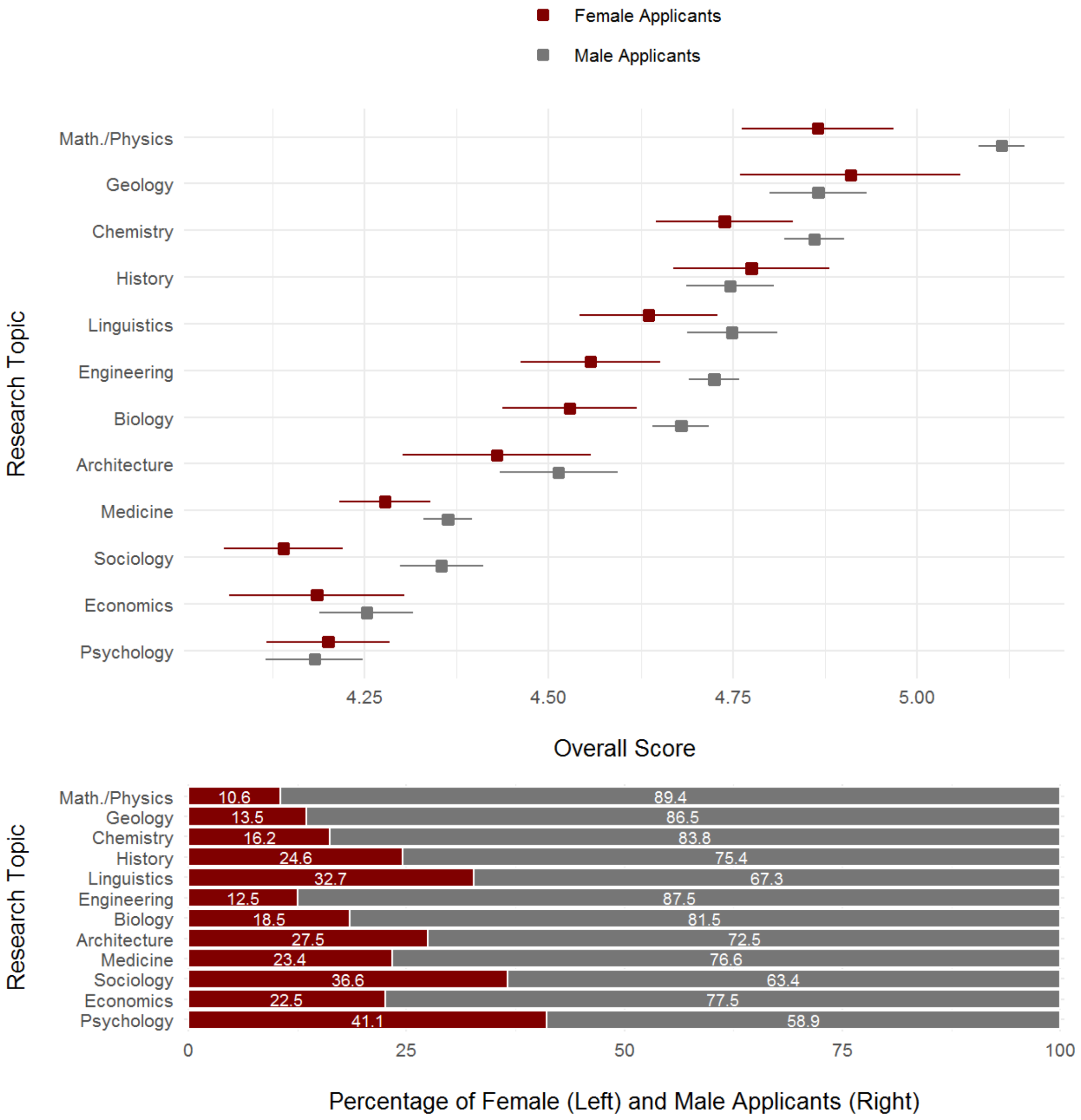

Upper panel: Average (mean) overall scores by research topic for female and male applicants; horizontal lines indicate Wald 95% confidence intervals. Lower panel: Gender distribution in the research topics.

For peer review only - http://bmjopen.bmj.com/site/about/guidelines.xhtml

Figure S2: Overall scores depending on the age and gender of the applicants.

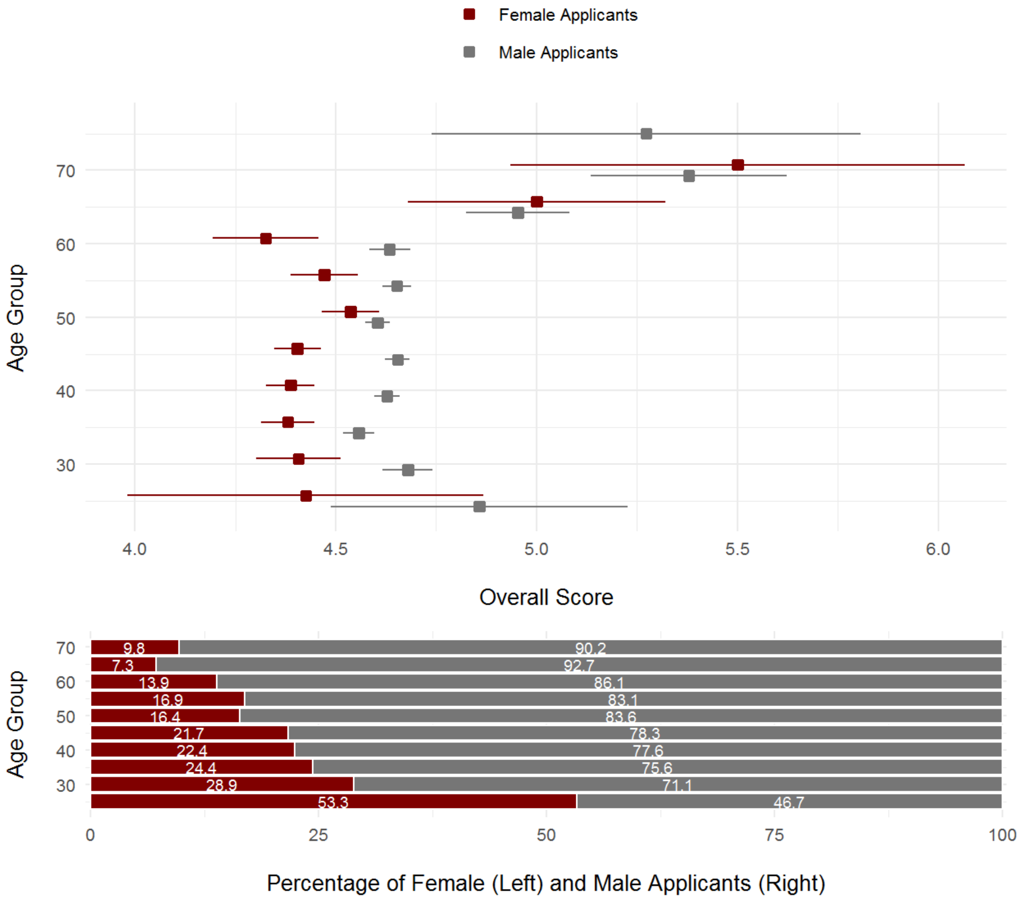

Upper panel: Average (mean) overall scores by five-year age group for female and male applicants; horizontal lines indicate Wald 95% confidence intervals. Lower panel: Gender distribution in the age groups.

For peer review only - http://bmjopen.bmj.com/site/about/guidelines.xhtml

Figure S3: Overall scores depending on the applicant's institution type and gender.

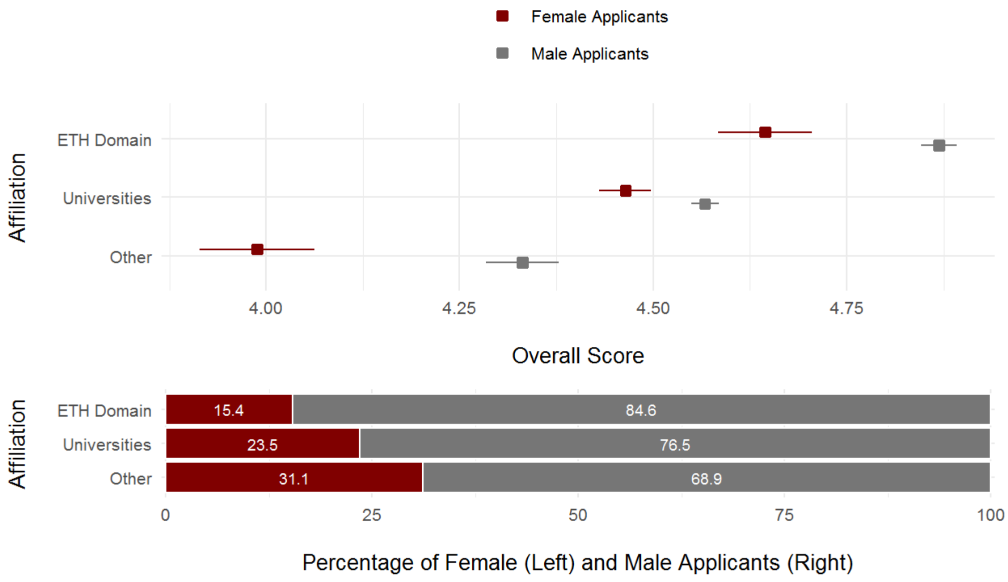

Upper panel: Average (mean) overall scores by affiliation times for female and male applicants; horizontal lines indicate Wald 95% confidence intervals. Lower panel: Gender distribution for the different affiliation types.

For peer review only - http://bmjopen.bmj.com/site/about/guidelines.xhtml

Figure S4: Average overall scores by nationality for female and male applicant; horizontal lines indicate Wald 95% confidence intervals.

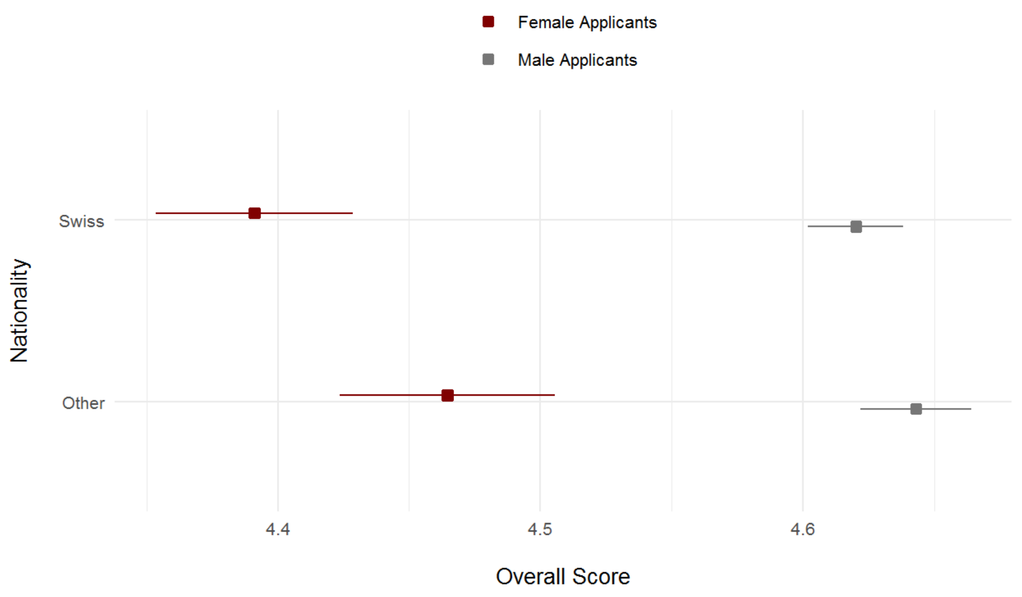

Figure S5: Average (mean) overall scores by application call deadline for female and male applicants; horizontal lines indicate Wald 95% confidence intervals.

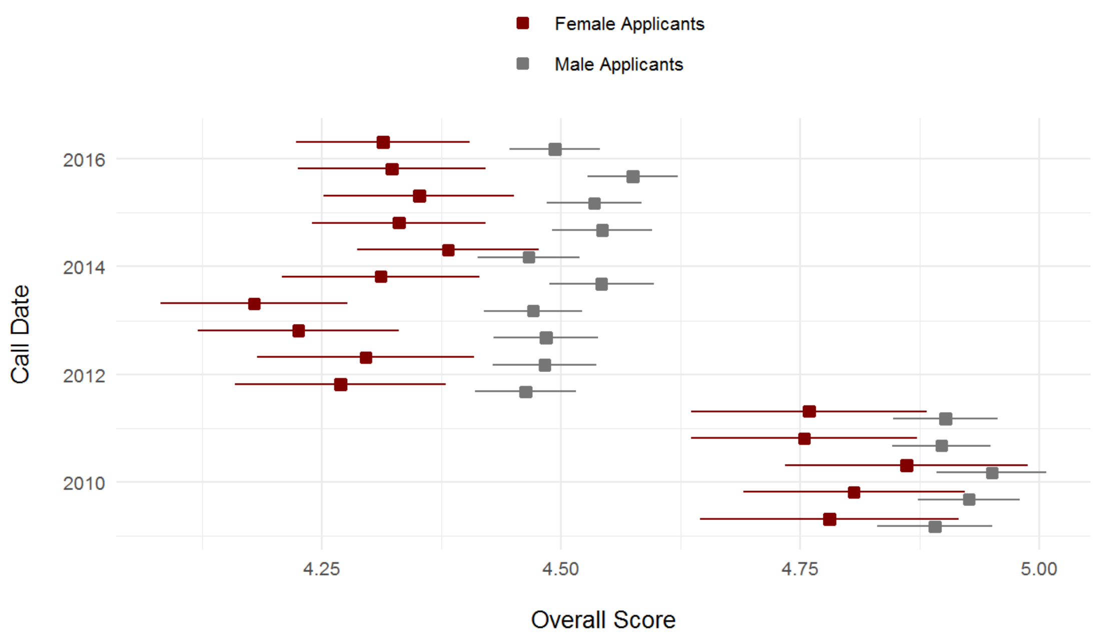

For peer review only - http://bmjopen.bmj.com/site/about/guidelines.xhtml

Text S1: Old and new evaluation forms. The new forms were implemented from the 1. October 2011 call onwards.

OLD FORM

1. Synopsis

	Excellent	Very Good	Good	Average	Satisfactory	Poor		Not considered
Current scientific interest and impact of the project								
Originality of the work								
Suitability and originality of the methods to be used								
Feasibility of the project								
Experience and past performance of the applicant								
Specific abilities of the applicants for the proposed project								

Overall assessment								

Comments regarding the overall assessment

2. Detailed evaluation

Current scientific interest and impact of the project

Originality of the work

For peer review only

Suitability and originality of the methods to be used

Feasibility of the project

Experience and past performance of the applicant

Specific abilities of the applicants for the proposed project

---

Other comments

For peer review only

For peer review only - http://bmjopen.bmj.com/site/about/guidelines.xhtml

NEW FORM

1. Synopsis

	outstanding	excellent	very good	good	average	poor		Not considered
Applicants' scientific track record and expertise								
Scientific relevance, originality and topicality								
Suitability of methods and feasibility								

Overall assessment								

Comments regarding the overall assessment

2. Detailed evaluation

Applicants' scientific track record and expertise

Scientific relevance, originality and topicality

Suitability of methods and feasibility

For peer review only

3. Further comments & declaration concerning conflicts of interests (will not be forwarded to applicants)

Confidential messages

The topic of the proposed project

Declaration concerning conflict of interests (comments, if applicable

For peer review only

STROBE Statement—Checklist of items that should be included in reports of ***cross-sectional studies***

	Item No	Recommendation	Page No
**Title and abstract**	1	(*a*) Indicate the study's design with a commonly used term in the title or the abstract	1
		(*b*) Provide in the abstract an informative and balanced summary of what was done and what was found	2
**Introduction**			
Background/rationale	2	Explain the scientific background and rationale for the investigation being reported	4-5
Objectives	3	State specific objectives, including any prespecified hypotheses	5
**Methods**			
Study design	4	Present key elements of study design early in the paper	6
Setting	5	Describe the setting, locations, and relevant dates, including periods of recruitment, exposure, follow-up, and data collection	5
Participants	6	(*a*) Give the eligibility criteria, and the sources and methods of selection of participants	n.a.
Variables	7	Clearly define all outcomes, exposures, predictors, potential confounders, and effect modifiers. Give diagnostic criteria, if applicable	6
Data sources/ measurement	8*	For each variable of interest, give sources of data and details of methods of assessment (measurement). Describe comparability of assessment methods if there is more than one group	6
Bias	9	Describe any efforts to address potential sources of bias	7
Study size	10	Explain how the study size was arrived at	6
Quantitative variables	11	Explain how quantitative variables were handled in the analyses. If applicable, describe which groupings were chosen and why	7
Statistical methods	12	(*a*) Describe all statistical methods, including those used to control for confounding	7
		(*b*) Describe any methods used to examine subgroups and interactions	7
		(*c*) Explain how missing data were addressed	7
		(*d*) If applicable, describe analytical methods taking account of sampling strategy	n.a.
		(*e*) Describe any sensitivity analyses	n.a.
**Results**			
Participants	13*	(a) Report numbers of individuals at each stage of study—eg numbers potentially eligible, examined for eligibility, confirmed eligible, included in the study, completing follow-up, and analysed	8
		(b) Give reasons for non-participation at each stage	n.a.
		(c) Consider use of a flow diagram	n.a.
Descriptive data	14*	(a) Give characteristics of study participants (eg demographic, clinical, social) and information on exposures and potential confounders	8
		(b) Indicate number of participants with missing data for each variable of interest	8
Outcome data	15*	Report numbers of outcome events or summary measures	8,9, Table 2

For peer review only - http://bmjopen.bmj.com/site/about/guidelines.xhtml

Main results	16	(*a*) Give unadjusted estimates and, if applicable, confounder-adjusted estimates and their precision (eg, 95% confidence interval). Make clear which confounders were adjusted for and why they were included	Table 3
		(*b*) Report category boundaries when continuous variables were categorized	n.a.
		(*c*) If relevant, consider translating estimates of relative risk into absolute risk for a meaningful time period	n.a.
Other analyses	17	Report other analyses done—eg analyses of subgroups and interactions, and sensitivity analyses	10
**Discussion**			
Key results	18	Summarise key results with reference to study objectives	11
Limitations	19	Discuss limitations of the study, taking into account sources of potential bias or imprecision. Discuss both direction and magnitude of any potential bias	13
Interpretation	20	Give a cautious overall interpretation of results considering objectives, limitations, multiplicity of analyses, results from similar studies, and other relevant evidence	11-14
Generalisability	21	Discuss the generalisability (external validity) of the study results	13
**Other information**			
Funding	22	Give the source of funding and the role of the funders for the present study and, if applicable, for the original study on which the present article is based	15

*Give information separately for exposed and unexposed groups.

Note: An Explanation and Elaboration article discusses each checklist item and gives methodological background and published examples of transparent reporting. The STROBE checklist is best used in conjunction with this article (freely available on the Web sites of PLoS Medicine at http://www.plosmedicine.org/, Annals of Internal Medicine at http://www.annals.org/, and Epidemiology at http://www.epidem.com/). Information on the STROBE Initiative is available at www.strobe-statement.org.

For peer review only

For peer review only - http://bmjopen.bmj.com/site/about/guidelines.xhtml

Gender and Other Potential Biases in Peer Review: Cross-sectional Analysis of 38,250 External Peer Review Reports

Journal:	*BMJ Open*
Manuscript ID	bmjopen-2019-035058.R1
Article Type:	Original research
Date Submitted by the Author:	10-Mar-2020

Complete List of Authors:	Severin, Anna; University of Bern, ISPM<br>Martins, Joao; European Research Council<br>Heyard, Rachel; Swiss National Science Foundation<br>Delavy, François; Swiss National Science Foundation<br>Jorstad, Anne; Swiss National Science Foundation<br>Egger, Matthias; University of Bern, Institute of Social & Preventive Medicine

**Primary Subject Heading**:	Epidemiology
Secondary Subject Heading:	Medical publishing and peer review
Keywords:	EPIDEMIOLOGY, STATISTICS & RESEARCH METHODS, Organisational development < HEALTH SERVICES ADMINISTRATION & MANAGEMENT

I, the Submitting Author has the right to grant and does grant on behalf of all authors of the Work (as defined in the below author licence), an exclusive licence and/or a non-exclusive licence for contributions from authors who are: i) UK Crown employees; ii) where BMJ has agreed a CC-BY licence shall apply, and/or iii) in accordance with the terms applicable for US Federal Government officers or employees acting as part of their official duties; on a worldwide, perpetual, irrevocable, royalty-free basis to BMJ Publishing Group Ltd ("BMJ") its licensees and where the relevant Journal is co-owned by BMJ to the co-owners of the Journal, to publish the Work in this journal and any other BMJ products and to exploit all rights, as set out in our licence.

The Submitting Author accepts and understands that any supply made under these terms is made by BMJ to the Submitting Author unless you are acting as an employee on behalf of your employer or a postgraduate student of an affiliated institution which is paying any applicable article publishing charge ("APC") for Open Access articles. Where the Submitting Author wishes to make the Work available on an Open Access basis (and intends to pay the relevant APC), the terms of reuse of such Open Access shall be governed by a Creative Commons licence – details of these licences and which Creative Commons licence will apply to this Work are set out in our licence referred to above.

Other than as permitted in any relevant BMJ Author's Self Archiving Policies, I confirm this Work has not been accepted for publication elsewhere, is not being considered for publication elsewhere and does not duplicate material already published. I confirm all authors consent to publication of this Work and authorise the granting of this licence.

For peer review only

Gender and Other Potential Biases in Peer Review: Cross-sectional Analysis of 38,250 External Peer Review Reports

Anna Severin[1,2], João Martins[1,3], Rachel Heyard[1], François Delavy[1], Anne Jorstad[1], Matthias Egger[1,2]

[1]Swiss National Science Foundation, Bern, Switzerland

[2] Institute of Social and Preventive Medicine, University of Bern, Bern, Switzerland

[3] European Research Council Executive Agency, Brussels, Belgium

Corresponding Author:

Professor Matthias Egger MD MSc FFPH

Swiss National Science Foundation

Wildhainweg 3

3001 Bern, Switzerland

matthias.egger@ispm.unibe.ch

For peer review only

Abstract 287 words, main text 3895 words, strengths and limitations 120 words, 1 figure, 3 tables, 31 references, supplementary materials with 1 table, 5 figures and 1 text.

For peer review only - http://bmjopen.bmj.com/site/about/guidelines.xhtml

ABSTRACT

Objectives: To examine whether the gender of applicants and peer reviewers and other factors influence peer review of grant proposals submitted to a national funding agency.

Setting: Swiss National Science Foundation (SNSF).

Design: Cross-sectional analysis of peer review reports submitted 2009 to 2016 using linear mixed effects regression models adjusted for research topic, applicant's age, nationality, affiliation and calendar period.

Participants: External peer reviewers.

Primary outcome measure: Overall score on a scale from 1 (worst) to 6 (best).

Results: Analyses included 38,250 reports on 12,294 grant applications from Medicine, Architecture, Biology, Chemistry, Economics, Engineering, Geology, History, Linguistics, Mathematics, Physics, Psychology and Sociology submitted by 26,829 unique peer reviewers. In univariable analysis, male applicants received more favourable evaluation scores than female applicants (+0.18 points; 95% CI 0.14-0.23), and male reviewers awarded higher scores than female reviewers (+0.11; 95% CI 0.08-0.15). Applicant-nominated reviewers awarded higher scores than reviewers nominated by the SNSF (+0.53; 95% CI 0.50-0.56), and reviewers from outside of Switzerland more favourable scores than reviewers affiliated with Swiss institutions (+0.53; 95% CI 0.49-0.56). In multivariable analysis, differences between male and female applicants were attenuated (to +0.08; 95% CI 0.04-0.13) whereas results changed little for source of nomination and affiliation of reviewers. The gender difference increased after September 2011, when new evaluation forms were introduced (P=0.033 from test of interaction).

Conclusions: Peer review of grant applications at SNSF might be prone to biases stemming from different applicant and reviewer characteristics. The SNSF abandoned the nomination of peer reviewers by applicants. The new form introduced in 2011 may inadvertently have given more emphasis to the applicant’s track record. We encourage other funders to conduct similar studies, in order to improve the evidence base for rational and fair research funding.

Keywords: peer review, bias, gender matching hypothesis, confounding, mixed effects models

For peer review only - http://bmjopen.bmj.com/site/about/guidelines.xhtml

STRENGTHS AND LIMITATIONS OF THIS STUDY

- This study was based on a large sample of peer review reports on project proposals from medicine and other disciplines submitted to the national Swiss funding agency.
- It is one of a few studies examining the interaction between gender of main applicant and gender of reviewers and the ‘gender matching hypothesis’, as well as the influence of other characteristics of applicants.
- This study only examined scores from peer review, but not the determinants of the final funding decision or the level of funding. It is therefore unclear whether the differences in scores analysed in the present study influenced funding decisions.
- This study was carried out by researchers affiliated with the funding agency and not by an independent group of researchers.

For peer review only - http://bmjopen.bmj.com/site/about/guidelines.xhtml

BACKGROUND

Expert peer review of research proposals is the accepted best practice for determining which projects are allocated funding (1). The legitimacy of funding decisions relies on a funder's ability to minimize bias in grant evaluations that results from factors that are unrelated to the quality of the applications (2). Empirical studies suggest that the evaluation of proposals is prone to biases that may relate to both applicant and reviewer characteristics (3)(4). Potential discrimination against women is the most frequently investigated bias (5). A meta-analysis of 21 studies published 1987 to 2004 found heterogeneous results, with overall a small gender difference in grant awards, with more men receiving grants than women (6). More recently, analyses of grant applications submitted to the Canadian Institutes of Health Research (CIHR) 2012 to 2014 showed that female applicants received lower scores (7) and had lower grant success (8). Similarly, a study of critiques of applications for renewal of National Institutes of Health (NIH) grants found that reviewers assigned significantly worse priority, approach, and significance scores to female than male principle investigators (9). Finally, the success rate of women applying for European Research Council (ERC) Starting Grants, Consolidator Grants or Advanced Grants 2007 to 2016 was consistently lower than the success rate of men (10).

Other factors than gender can influence peer review. A study of the Australian Research Council found that applicant-nominated reviewers tended to give better ratings than panel-nominated reviewers (11). Further, an analysis of data from the Austrian Science Fund suggested that international peer reviewers affiliated with research institutions located in countries known for high scientific productivity were generally more stringent than national reviewers (12).

The Swiss National Science Foundation (SNSF) supports basic research and use-inspired basic research in all disciplines. The main funding scheme of the SNSF is project funding, which provides support to independent researchers who propose research on self-chosen topics (13). The proposals submitted to the SNSF are peer reviewed by at least two external experts. The foundation allowed grant applicants to suggest reviewers to evaluate submissions via a “positive list”. The names put forward on the list were then considered as potential reviewers, after a careful check for conflicts of interest (CoI). The SNSF frequently invites reviewers from abroad to review grant applications. Of note, the SNSF introduced new evaluation forms and guidelines for peer reviewers in September 2011, which we describe in the Methods section.

For peer review only - http://bmjopen.bmj.com/site/about/guidelines.xhtml

To gain insights into gender bias and other potential biases in peer review, we analysed the database of the SNSF to examine the determinants of overall scores from external peer reviewers in project funding.

METHODS

Evaluation of Grant Applications at the SNSF

The evaluation consists of four steps (13). The administrative office first checks eligibility and assigns grant applications to two members of the National Research Council (referee and co-referee) based on their field of expertise. Second, eligible proposals are peer-reviewed by external experts. External reviewers were identified in several ways: (i) grant applicants suggested experts via the "positive list", (ii) the referee of the National Research Council suggested reviewers, (iii) the SNSF administrative offices proposed experts, and (iv) experts who declined to review suggested other reviewers (13). For each application, at least two external reviews were required.

The final choice of reviewers was made by the SNSF. Reviewers from the positive list were chosen only if they had the required expertise and there were no CoI. To exclude any CoI, SNSF employees checked whether reviewers had submitted an application for the same call, whether they had published with the applicants in the past five years and whether they work at the same institution or in a closely associated unit. Applicants could also submit a "negative list" of reviewers who, because of a possible CoI, should not be contacted. Providing a positive or a negative list was optional and the lists could include one or several names.

The peer review forms and assessment scale were changed in September 2011 to simplify the review, and to achieve a more equal distribution of scores, with fewer proposals in the top category. Up to September 2011, peer reviewers were asked to score six criteria: i) current scientific interest and impact of the project; ii) originality of the work; iii) suitability of the methods; iv) work plan, feasibility, cost; v) experience and past performance of the applicants; and vi) specific abilities of the investigators for the proposed project. Reviewers were asked to "give a rating and provide explanatory comments" for each of the six criteria. In September 2011, new evaluation forms were introduced (13)(14), which asked experts to

For peer review only - http://bmjopen.bmj.com/site/about/guidelines.xhtml

review proposals according to three criteria: i) the applicants' scientific track-record and expertise; ii) the scientific relevance, originality and topicality of the proposed research and, in the case of use-inspired research, the research's broader impact; and iii) the suitability of the methods and feasibility. Furthermore, peer reviewers were asked to declare any CoI, and given the opportunity to submit confidential comments, which would not be seen by the applicants. Up to September 2011, reviewers scored the overall proposal and each criterion on a scale from 1 to 6: poor (score 1), satisfactory, average, good, very good, and excellent (score 6). In September 2011 the scale was changed to poor (score 1), average, good, very good, excellent, and outstanding (score 6). The two versions of the peer review form are reproduced in supplementary Text S1. The overall score was attributed by the external reviewers and there were no guidelines on how they should weight the criteria. Applications were not blinded and reviewers were therefore aware of applicant's gender and their track records.

In the third step of the evaluation, the two members of the Council (referee and co-referee) assessed the peer review reports and considered them when ranking the application relative to other proposals. In the fourth and final step, referee and co-referee presented their assessment at the meeting of the corresponding section of Council. Each application was then voted on and approved or rejected (13).

Data and Variables

We analysed the overall scores of external peer review reports submitted from 2009 to 2016. The outcome variable of interest was the overall score of a grant application given by external reviewers. Explanatory variables included meta-data on principle applicants and external peer reviewers, including source of reviewer (applicant-nominated vs. SNSF-nominated), gender of the applicant and gender of the reviewer (female vs. male) and country of affiliation of the reviewer (Switzerland vs. other). The mean ratio of female to male reviewers per grant application was 0.2. Eighteen percent of the grant applications had male-only external reviewers while only 1% had female-only external reviewers. SNSF-nominated experts included reviewers who were proposed by the referee, the SNSF office or by experts who declined to review. We also considered the research topic of a grant application as defined by the applicant when submitting their application (see Table S1 in the Supplement), type of institutional affiliation (which included Swiss Federal Institutes of

For peer review only - http://bmjopen.bmj.com/site/about/guidelines.xhtml

Technology and associated institutions, i.e. the ETH domain; Cantonal university; and other) and age of the applicant. Finally, we introduced a dummy variable to group applications submitted before and after September 2011.

Statistical Analysis

We used a linear mixed effects model to examine the effect of explanatory variables on the overall peer-review scores (15). This model was chosen because the data are clustered and hierarchical (16). Grant applications received two or more independent reviews, some reviewers had reviewed more than one application and many applicants had submitted more than one grant application over the study period, causing evaluation scores to be clustered at the levels of research projects, reviewers and applicants. We therefore introduced random intercepts for the identifiers of the reviewer, the applicant and the project in the model, thus taking into account the dependence between clustered scores (17). Given that y_{ijk} is the overall score given by reviewer *i* to application *j* submitted by applicant *k*, the final model is the following:

$$y_{ijk} = X_{ijk}\ beta + u_i + v_j + w_k + epsilon$$

where X_{ijk} is the matrix with the explanatory variables, *beta* is the regression coefficient vector, and u_i, v_j, w_k are the respective vectors of random intercepts and *epsilon* is the vector of random errors.

We ran crude and adjusted models. The latter were adjusted for gender of the applicant and reviewer, source of reviewers, country of affiliation of the reviewer, the applicant's age (per 10 year increase), affiliation, and nationality (Swiss vs. other), the field of research (12 categories), and the period of submission of the proposal (before or after the change in peer review forms and scale). To make adjusted and crude estimates comparable, we performed a complete case analysis by deleting peer review reports with missing values for any of the relevant variables. We examined interactions between the gender of the applicant and the gender of the reviewer, and other variables, by including interaction terms in the linear mixed models. We thus examined the 'gender matching hypothesis', which stipulates that female peer reviewers give higher scores to female researchers and that male reviewers do the same for male applicants (18). We used likelihood ratio tests to assess the strength of the evidence for interactions.

For peer review only - http://bmjopen.bmj.com/site/about/guidelines.xhtml

We present crude and adjusted regression coefficients, which reflect differences in peer review scores with their 95% confidence interval (CI). The notebook of the analysis, including summaries of the different statistical models, is available online at www.git.io/fhaJx.

Patient and Public Involvement

This analysis was based on peer review reports submitted to a national research funder. No patients were involved in developing the research question, outcome measures and overall design of the study. Due to the anonymous nature of the data, we were unable to disseminate the results of the research directly to study participants.

RESULTS

We analysed the summary scores of 38,250 external peer review reports on 12,294 project grant applications across all disciplines that were submitted from 2009 to 2016 by 26,829 unique reviewers from Switzerland and abroad. The average number of reviews per grant application was 3.1, applicants submitted an average of 2.1 grant applications and reviewers reviewed an average of 1.4 applications. The complete case mixed effects regression analyses were based on 37,989 reviews (99.3%).

Applicant characteristics

The 12,294 proposals were submitted by 5,820 applicants: 4,514 (77.6%) men and 1,306 (22.4%) women (Table 1). Most applicants were based at Cantonal universities, were Swiss, and the largest number was from medicine. Female applicants were younger than men and more likely to be affiliated with other institutions (for example universities of applied sciences, the arts or teacher education) than with the Federal ETH domain or the Cantonal universities. Women were also more likely to work in medicine, the social sciences and humanities (psychology, sociology, linguistics) than in STEM disciplines (Science, Technology, Engineering, and Mathematics) or biology (Table 1).

Peer review scores across groups of applicants and reviewers

Distributions of overall peer review scores were somewhat skewed, with applications more frequently being awarded high evaluation scores than low scores (see notebook at www.git.io/fhaJx). Male principle applicants received higher evaluation scores than female

For peer review only - http://bmjopen.bmj.com/site/about/guidelines.xhtml

principle applicants (Table 2). Similarly, the analysis of evaluation scores by gender of the reviewer showed that male reviewers tended to award higher scores than female reviewers. Applicant-nominated reviewers awarded higher scores than SNSF-nominated reviewers, and reviewers affiliated with institutions outside Switzerland awarded higher evaluation scores than reviewers affiliated with Swiss institutions.

There were important differences in evaluation scores across research fields. Grant applications in the natural and technical sciences or in linguistics and history received higher evaluation scores than applications from medicine, sociology or psychology (supplementary Figure S1). Gender differences in scores were more pronounced for some research topics (for example mathematics and physics and engineering, biology and medicine, sociology) than others (for example geology, history, psychology). Female applicants were underrepresented (below 50 percent) in all research topics (lower panel of supplementary Figure S1).

Applicants aged 60 years or older received the highest evaluation scores, independent of their gender. For the younger age groups, female applicants consistently received lower evaluation scores than male applicants (supplementary Figure S2). Female applicants were under represented across all age groups, except for the youngest age group, and representation was particularly low in older age groups (lower panel of supplementary Figure S2). Applications submitted by applicants affiliated with the ETH Domain received higher evaluation scores than applications from Cantonal universities or from other research institutions. Gender differences in scores were evident for all three affiliations, and women were under represented for all affiliations (supplementary Figure S3).

Grant applications submitted by Swiss applicants received slightly lower scores than those submitted by applicants with other nationalities, with a similar gap between genders (supplementary Figure S4). Finally, supplementary Figure S5 shows that, as expected, applications submitted before the new forms were introduced received higher scores than applications evaluated later.

Linear Mixed-Effects Models

Table 3 shows crude and adjusted differences in peer review scores by characteristics of applicants, reviewers and research proposals. In the crude model, the difference between male and female applicants was 0.18 points favouring men. More substantial differences of

For peer review only - http://bmjopen.bmj.com/site/about/guidelines.xhtml

0.53 points were observed for source of reviewer (0.53 points higher if the reviewer was nominated by the applicants) and country of affiliation of the reviewer (0.53 higher for reviewers from outside Switzerland). Substantial differences were also observed across disciplines. For example, scores were on average 0.68 points higher in mathematics and physics than in medicine, but 0.12 point lower in psychology than in medicine (Table 3). Compared to crude differences, most adjusted differences were smaller. For example, the adjusted difference between male and female applicants was reduced from 0.18 to 0.08 points. One exception was the difference observed between proposals evaluated before or after the introduction of the new peer review forms in September 2011 (0.43 points higher scores before the introduction in both analyses).

Interactions between gender of the applicants and other variables

We examined possible interactions between the genders of the applicants with the other fixed-effect variables in the model shown in Table 2. In other words, we examined whether the differences observed between female and male applicants varied across the levels of the other variables. We found that male reviewers gave higher scores both to male and female applicants than female reviewers, but this difference was considerably greater for male than for female applicants. Figure 1 shows the predicted values of the overall score from the bivariable model (P=0.011 from test of interaction). There was some evidence that the gender difference in scores became larger after the introduction of the new evaluation form (P=0.065, Figure 1). There was also strong evidence for an interaction (P<0.0001) between gender of the first applicant and his or her affiliation: the gender differences in scores were smallest for applicants based at one of the Cantonal universities, larger for the ETH domain and most pronounced for other institutions of higher education (for example universities of applied sciences, the arts or teacher education, see Figure 1). The interaction P values from the adjusted models were 0.037 (gender of peer reviewer), 0.003 (affiliation of applicant) and 0.033 (change of evaluation form). All P values from the bivariable and multivariable interaction tests are shown in supplementary Table S2. Interaction effects were small. The effect sizes can be found in the online notebook at www.git.io/fhaJx.

For peer review only - http://bmjopen.bmj.com/site/about/guidelines.xhtml

DISCUSSION

This study of 38,250 distinct grant reviews of 12,294 proposals across all disciplines, which were submitted to the SNSF between 2009 and 2016 by 5,832 applicants is to the best of our knowledge one of the largest studies of peer review reports on research proposals ever conducted. Female applicants received lower scores than male applicants. The gender difference was attenuated in multivariable analysis: it was partly explained by the fact that women were under represented among applicants in the fields and institutions whose proposals were rated highly, for example mathematics and physics, and institutions of the ETH domain. Our finding is in line with a text analysis of critiques of funded and unfunded National Institutes of Health (NIH) grant applications, which found that reviewers assigned significantly worse scores for research approach, significance and priority to female than male applicants. The authors concluded that reviewers implicitly hold male and female applicants to different standards of evaluation (19)

Although a substantial proportion of the gender gap in our study was explained by other factors, these factors might be a reflection of the leaky pipeline, i.e. “the phenomenon of women dropping out of research and academic careers at a faster rate than men” (20), which is well documented for Switzerland (21,22). The academic pipeline in Switzerland is particularly leaky in the life sciences, social sciences and humanities. In STEM the rate of dropout of women is less pronounced, but they are a minority from the start: among PhD students only about 20% are women, whereas in the social sciences, humanities, and the life sciences the majority of doctoral students are women (22).

A noteworthy finding of our study was the interaction between the gender of applicants and peer reviewers. In contrast to Jayasinghe and colleagues (18), who analysed 7,153 reviewer ratings at the Australian Research Council large grant programme and other smaller studies (2)(23), we found evidence supporting the ‘gender matching hypothesis’. Male reviewers gave systematically higher ratings to male applicants than to female applicants, whereas the same phenomenon could not be observed for female reviewers. If such matching bias was present, male reviewers will have favoured male applicants, despite the fact that the proposals from male and female applicants were of similar quality. Alternatively, assuming proposals from male applicants were in fact stronger, female reviewers could have been biased against men and could have downgraded their proposals.

For peer review only - http://bmjopen.bmj.com/site/about/guidelines.xhtml

Male reviewers may have given more weight to the track record of applicants than female reviewers. In this context, it is interesting that the gender gap became wider after September 2011, when new evaluation forms for external peer review were introduced. The new guidelines and form separated the criteria related to the applicants, and the criteria related to the proposed project. On the new form, the applicant's track record was the first criterion out of a total of three, whereas it was the fifth out of six criteria on the old form. Although this was not intended, the reform may have led to reviewers giving more weight to the track record of applicants, due to its prominence on the new form. Commenting on a Canadian study, which showed that the gender gap in grant funding was due to less positive assessments of women as principal investigators whereas the quality of the proposed research was similar for women and men (24), Raymond and Goodman asked funders to "evaluate projects, not people" (25). We are planning additional analyses to examine whether at the SNSF the same phenomenon is at play, i.e. whether the gender gap is driven by the assessments of the track record. Furthermore, the SNSF is discussing changes to the peer review form.

For peer review only

Our results confirm those from the Australian Research Council, which showed that applicant-nominated reviewers gave higher ratings than panel-nominated reviewers (11). A study of peer review in biomedical journals also found that author-nominated reviewers submitted more favourable recommendations than editor-nominated reviewers (26). This difference may be interpreted in several ways. First, nominated reviewers may have a CoI that remained undetected in the SNSF CoI examination. Alternatively, applicants may nominate reviewers who are more familiar with their field than reviewers nominated by the SNSF, and thus more able to recognize the impact and importance of the proposed research. Like the Australian Research Council, the SNSF felt that bias was the more likely explanation and decided to discontinue the use of the "positive list" in 2016. Of note, applicants can still submit a "negative list" of reviewers that should not be used because of perceived CoI.

The gender effect was larger for proposals affiliated with an institution from the Federal ETH domaine, and especially, from other institutions (for example universities of applied sciences, the arts or teacher education) compared to applicants affiliated to Cantonal universities. In this context, male applicants from other Institutions got systematically higher ratings than their female peers, while the observed gender differences in scores for applicants from Cantonal universities was less pronounced, especially after adjustment for

confounding variables. The underrepresentation of female researchers in the ETH domain and in other institutions might have contributed to this situation, by making the few women applicants appear less qualified to the male reviewers.

Peer reviewers affiliated with a Swiss research institution gave lower scores than reviewers from outside Switzerland. A study of the Austrian Science Fund suggested that reviewers from countries with high scientific productivity were more stringent than national reviewers (12). Switzerland belongs to the most productive countries in terms of research output (27) and this might explain why reviewers affiliated with Swiss research institutions award lower evaluation scores than reviewers from abroad. In contrast to the Austrian study (12), the Australian data showed that reviewers affiliated with an institution in the United States of America (USA) were more lenient than reviewers affiliated with institutions located in the United Kingdom, Germany or Australia (28), despite the fact that the USA is the country with the highest research output globally (27). Other explanations for the lower scores awarded by Swiss reviewers include greater knowledge of the local research capacity and expertise, or bias, if reviewers based in Switzerland downgraded the proposals of their competitors.

Our study has several limitations. First, we did not examine the determinants of the final funding decision or the level of funding. It is therefore unclear whether the differences in scores analysed in the present study influenced funding decisions. Such analyses are planned for the future. Second, this is an observational study and it is therefore difficult to infer causality from the associations observed. Chance, bias, and confounding variables must be considered as possible explanations for associations between reviewer and applicant characteristics and overall scores (29). We tried to control for confounding by adjusting for these variables in regression models. We are considering randomized experiments to test certain interventions (e.g., blinding) in order to prevent or reduce gender effects for the future. Third, our results are relevant to the Swiss context, but may not be applicable to other countries. Fourth, we did not attempt to rate the expertise of the reviewers, and adjust for the differences in individual reviewers scores based on their previous performance. We also did not measure the scientific productivity of applicants, and adjust scores for productivity. Other studies have shown that women have lower productivity than men (7,30). Fifth, this study was carried out by researchers affiliated with the SNSF and not by an independent research institution. As studies might be influenced by the expectations of the researchers of the study, the credibility of the results might be reduced. We address this by making the data available for

For peer review only - http://bmjopen.bmj.com/site/about/guidelines.xhtml

replication. Finally, we examined project funding only, but not career funding or programme funding.

CONCLUSIONS

In conclusion, our results had important implications for the evaluation of project grant proposals at the SNSF. The foundation abandoned the nomination of peer reviewers by applicants, and made members of evaluation panels aware of the other factors, including the gender and affiliation of reviewers, that can influence review scores. We encourage all funding bodies to contribute to research on potential biases in research funding, and ways of preventing them (31).

For peer review only

For peer review only - http://bmjopen.bmj.com/site/about/guidelines.xhtml

FOOTNOTES

Acknowledgements: Earlier results from this analysis were presented at the 5th International Congress on Peer Review and Scientific Publication, Chicago, Illinois, USA; September 10-12, 2017. We are grateful to Angelika Kalt, Benjamin Rindlisbacher, Barbara Curdy-Korrodi and two expert reviewers for helpful comments on previous versions of this paper, and to Andreas Limacher and Lukas Bütikofer (Clinical Trials Unit of the Faculty of Medicine of the University of Bern) for advice on the statistical analyses.

Contributors: AS, JM and ME conceived the study. JM and RH performed statistical analyses. FD and AJ contributed to data management and statistical analyses. AS and JM wrote the first draft of the paper, which was revised by ME, AS and RH. All authors contributed to and approved the final version.

Funding: This work was supported by the SNSF (internal funds and grant number 174281).

Competing interests: The authors are employees of the SNSF; they declare that they have no other competing interests.

Patient consent: Not applicable.

Ethics approval: Under Swiss law, not ethics approval is required for this type of study. Peer reviewers did not provide consent. No peer reviewer, applicant or proposal can be identified from this report.

Data sharing statement: The data analysed in this study are available to others on request for an approved research project, after signing a data sharing agreement.

For peer review only

References

1. Harman G. The Management of Quality Assurance: A Review of International Practice. Higher Education Quarterly 52(4):345 - 364. 2002;52(4):345–64.

2. Bornmann L, Daniel H-D. Gatekeepers of science—Effects of external reviewers' attributes on the assessments of fellowship applications. Journal of Informetrics. 2007 Jan;1(1):83–91.

3. Bornmann L, Daniel H-D. Gatekeepers of science - Effects of external reviewers' attributes on the assessments of fellowship applications. J Informetr. 2007 Jan;1(1):83–91.

4. Demicheli V, C DP. Peer review for improving the quality of grant applications (Review). 2008;(2).

5. Mutz R, Bornmann L, Daniel H-D. Does Gender Matter in Grant Peer Review? An Empirical Investigation Using the Example of the Austrian Science Fund. Z Psychol-J Psychol. 2012;220(2):121–9.

6. Bornmann L, Mutz R, Daniel H. Gender differences in grant peer review: A meta-analysis. Journal of Informetrics. 2007 Jul;1(3):226–238.

7. Tamblyn R, Girard N, Qian CJ, Hanley J. Assessment of potential bias in research grant peer review in Canada. Can Med Assoc J. 2018 Apr 23;190(16):E489–99.

8. Burns KEA, Straus SE, Liu K, Rizvi L, Guyatt G. Gender differences in grant and personnel award funding rates at the Canadian Institutes of Health Research based on research content area: A retrospective analysis. PLoS Med. 2019;16(10):e1002935.

9. Kaatz A, Lee Y-G, Potvien A, Magua W, Filut A, Bhattacharya A, et al. Analysis of National Institutes of Health R01 Application Critiques, Impact, and Criteria Scores: Does the Sex of the Principal Investigator Make a Difference? Acad Med. 2016;91(8):1080–8.

10. Bautista-Puig N, Garcia-Zorita C, Mauleon E. European Research Council: excellence and leadership over time from a gender perspective. Res Evaluat. 2019 Oct;28(4):370–82.

11. Marsh HW, Bonds NW, Jayasinghe UW. Peer review process: Assessments by applicant-nominated referees are biased, inflated, unreliable and invalid. Aust Psychol. 2007 Mar;42(1):33–8.

12. Fischer C, Reckling FJ. Factors Influencing Approval Probability in Austrian Science Fund (FWF) Decision-Making Procedures - FWF Stand-Alone Projects Programme, 1999 to 2008 [Internet]. Rochester, NY: Social Science Research Network; 2010 Dec [cited 2019 May 5]. Report No.: ID 1725985. Available from: https://papers.ssrn.com/abstract=1725985

13. Swiss National Science Foundation. Funding Regulations. Regulations of the Swiss National Science Foundation on research grants. Version 1.1.2016 [Internet]. 2016. Available from: http://www.snf.ch/SiteCollectionDocuments/allg_reglement_16_e.pdf

For peer review only - http://bmjopen.bmj.com/site/about/guidelines.xhtml

14. Project funding - SNF [Internet]. [cited 2019 May 25]. Available from: http://www.snf.ch/en/theSNSF/evaluation-procedures/project-funding/Pages/default.aspx

15. Bates D, Maechler M, Bolker BM, Walker SC. Fitting Linear Mixed-Effects Models Using lme4. J Stat Softw. 2015 Oct;67(1):1–48.

16. Jayasinghe UW, Marsh HW, Bond N. A multilevel cross-classified modelling approach to peer review of grant proposals: the effects of assessor and researcher attributes on assessor ratings. Journal of the Royal Statistical Society: Series A (Statistics in Society). 2003 Oktober;166(3):279–300.

17. Harrison XA, Donaldson L, Correa-Cano ME, Evans J, Fisher DN, Goodwin CED, et al. A brief introduction to mixed effects modelling and multi-model inference in ecology. PeerJ. 2018;6:e4794.

18. Jayasinghe UW, Marsh HW, Bond N. A multilevel cross-classified modelling approach to peer review of grant proposals: the effects of assessor and researcher attributes on assessor ratings. J Royal Statistical Soc A. 2003 Oct;166(3):279–300.

19. Kaatz A, Lee Y-G, Potvien A, Magua W, Filut A, Bhattacharya A, et al. Analysis of National Institutes of Health R01 Application Critiques, Impact, and Criteria Scores: Does the Sex of the Principal Investigator Make a Difference? Academic Medicine. 2016 Aug;91(8):1080–8.

20. Directorate-General for Research and Innovation. She Figures 2015 [Internet]. 2015. 224 p. Available from: https://ec.europa.eu/research/swafs/index.cfm?pg=library&lib=gender_equality

21. Bataille P, Le Feuvre N, Morales SK. Should I stay or should I go? The effects of precariousness on the gendered career aspirations of postdocs in Switzerland. Eur Educ Res J. 2017 May;16(2–3):313–31.

22. Schubert F, Engelage S. Wie undicht ist die Pipeline? Wissenschaftskarrieren von promovierten Frauen. Köln Z Soziol. 2011 Sep;63(3):431–57.

23. Sonnert G. What Makes a Good Scientist?: Determinants of Peer Evaluation among Biologists. Social Studies of Science. 1995;25(1):35–55.

24. Witteman HO, Hendricks M, Straus S, Tannenbaum C. Are gender gaps due to evaluations of the applicant or the science? A natural experiment at a national funding agency. The Lancet. 2019 Feb;393(10171):531–40.

25. Raymond JL, Goodman MB. Funders should evaluate projects, not people. The Lancet. 2019 Feb;393(10171):494–5.

26. Schroter S, Tite L, Hutchings A, Black N. Differences in review quality and recommendations for publication between peer reviewers suggested by authors or by editors. JAMA-J Am Med Assoc. 2006 Jan 18;295(3):314–7.

27. Country outputs | Nature Index [Internet]. [cited 2019 May 14]. Available from: https://www.natureindex.com/country-outputs

For peer review only - http://bmjopen.bmj.com/site/about/guidelines.xhtml

28. Wood F. The peer review process [Internet]. Canberra: Australian Govt. Pub. Service; 1997 [cited 2019 May 5] p. 189. Available from: https://trove.nla.gov.au/version/45600880

29. Smith GD, Ebrahim S. Data dredging, bias, or confounding - They can all get you into the BMJ and the Friday papers. Br Med J. 2002 Dec 21;325(7378):1437-+.

30. Wennerås C, Wold A. Nepotism and sexism in peer-review. Nature. 1997 May;387(6631):341–3.

31. Tricco AC, Thomas SM, Antony J, Rios P, Robson R, Pattani R, et al. Strategies to Prevent or Reduce Gender Bias in Peer Review of Research Grants: A Rapid Scoping Review. PLoS One. 2017 Jan 6;12(1):e0169718.

For peer review only

For peer review only - http://bmjopen.bmj.com/site/about/guidelines.xhtml

Legend to Figure

Figure 1: Gender differences in external evaluation scores by gender of the expert reviewer, affiliation and period of submission of the proposal.

Predicted values from bivariable, unadjusted models (upper panel) and the multivariable analysis (lower panel) are shown, together with their 95% confidence intervals. Scores range from 1 (worst) to 6 (best). Average (mean) overall scores are shown, horizontal lines indicate Wald 95% confidence intervals.

For peer review only

Table 1. Characteristics of applicants who submitted grant applications to the Swiss National Science Foundation between 2009 and 2016, stratified by gender.

	**Male applicants (n = 4,514, 78%)**	**Female applicants (n = 1,306, 22%)**
Age (mean, SD)	48.24 (8.63)	46.23 (8.27)
Affiliation		
ETH Domain	1,195 (26%)	219 (17%)
Other	481 (11%)	224 (17%)
Universities (reference)	2,838 (63%)	863 (66%)
Nationality		
Other than Swiss	1,896 (42%)	573 (44%)
Swiss	2,618 (58%)	733 (56%)
Field of research		
Medicine (reference)	1,029 (23%)	317 (24%)
Architecture	146 (3%)	56 (4%)
Biology	611 (14%)	129 (10%)
Chemistry	378 (8%)	76 (6%)
Economics	290 (6%)	84 (6%)
Engineering	527 (12%)	74 (6%)
Geology	144 (3%)	24 (2%)
History	209 (5%)	68 (5%)
Linguistics	203 (5%)	102 (8%)
Mathematics / Physics	491 (11%)	56 (4%)
Psychology	223 (5%)	164 (13%)
Sociology	263 (6%)	156 (12%)

The characteristics refer to the first submission of a project grant proposal during the study period. Numbers (%) are shown unless otherwise indicated. Analysis based on 5,820 unique applicants without missing values.

For peer review only - http://bmjopen.bmj.com/site/about/guidelines.xhtml

Table 2: Mean of overall score by groups of applicants and peer reviewers.

Group	No. of peer review reports	Mean overall score (SD)
Female Applicants	7,764	4.42 (1.25)
Male Applicants	30,455	4.63 (1.22)
Female Reviewers	7,591	4.44 (1.26)
Male Reviewers	30,659	4.63 (1.22)
Applicant-nominated Reviewers	8,755	5.12 (1.00)
SNSF-nominated Reviewers	29,495	4.43 (1.25)
International-based Reviewers	29,423	4.71 (1.19)
National-based Reviewers	8,604	4.16 (1.28)

SD, standard deviation.

Results based on 38,250 peer review reports.

For peer review only

Table 3: Crude and adjusted differences in external peer review evaluation scores by characteristics of applicants, reviewers and research proposals.

Variable	Number of reviews analysed	Unadjusted difference (95% CI)	P	Adjusted difference (95% CI)	P
Gender of the applicant			<0.001		<0.001
Male	30,263	0.18 (0.14 – 0.23)		0.08 (0.04 – 0.13)	
Female	7,716	0		0	
Gender of the reviewer			<0.001		<0.001
Male	30,442	0.11 (0.08 – 0.15)		0.08 (0.05 – 0.11)	
Female	7,537	0		0	
Source of nomination of reviewer			<0.001		<0.001
Applicant	8,688	0.53 (0.50 – 0.56)		0.49 (0.46 – 0.51)	
Office	29,291	0		0	
Country of affiliation of reviewer			<0.001		<0.001
Outside Switzerland	29,384	0.53 (0.49 – 0.56)		0.47 (0.44 – 0.50)	
Switzerland	8,595	0		0	
Age of the applicant	37,989		<0.001		<0.001
Per 10 year increase		0.06 (0.03 – 0.08)		0.05 (0.03 – 0.07)	
Affiliation of the applicant			<0.001		<0.001
ETH Domain	9,960	0.30 (0.26 – 0.34)		0.11 (0.07 – 0.16)	
Other	4,075	-0.24 (-0.30 - -0.19)		-0.19 (-0.25 – -0.14)	
Universities	23,944	0		0	
Nationality of the applicant			0.155		0.143
Other than Swiss	16,545	0.03 (-0.01 – 0.06)		-0.03 (-0.06 – 0.01)	
Swiss	21,434	0		0	
Field of research			<0.001		<0.001
Medicine	7,540	0		0	
Architecture	1,391	0.13 (0.03 – 0.24)		0.15 (0.05 – 0.25)	
Biology	3,872	0.30 (0.24 – 0.36)		0.27 (0.21 – 0.33)	
Chemistry	3,244	0.46 (0.39 – 0.53)		0.24 (0.17 – 0.31)	
Economics	2,171	-0.09 (-0.17 – -0.01)		-0.01 (-0.09 – 0.06)	
Engineering	4,880	0.32 (0.25 – 0.38)		0.07 (0.00 – 0.13)	
Geology	1,167	0.50 (0.39 – 0.60)		0.25 (0.14 – 0.35)	
History	2,053	0.35 (0.27 – 0.44)		0.32 (0.24 – 0.40)	
Linguistics	2,244	0.30 (0.22 – 0.38)		0.26 (0.18 – 0.34)	
Mathematics / Physics	3,979	0.68 (0.62 – 0.75)		0.45 (0.39 – 0.52)	
Psychology	2,458	-0.12 (-0.20 – -0.05)		-0.08 (-0.15 – 0.00)	
Sociology	2,980	-0.06 (-0.13 – 0.02)		0.01(-0.06 – 0.08)	
Introduction of reviewer guidelines			<0.001		<0.001
Before introduction	11,151	0.44 (0.41 – 0.47)		0.43 (0.40 – 0.46)	
After introduction	26,828	0		0	

95% CI, 95% confidence interval. Results from linear mixed effects models based on 37,979 complete peer review reports.

For peer review only - http://bmjopen.bmj.com/site/about/guidelines.xhtml

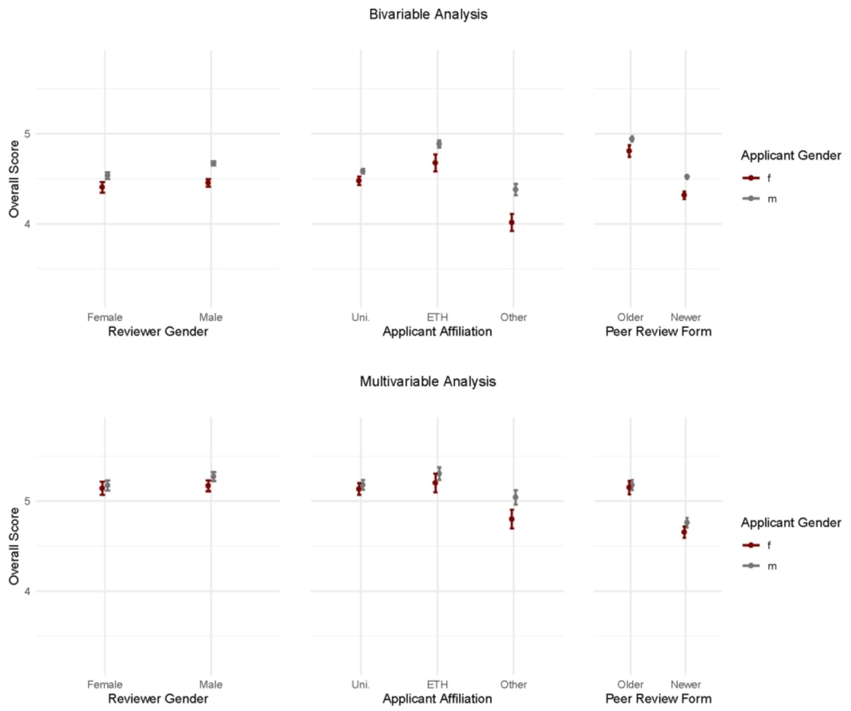

Gender differences in external evaluation scores by gender of the expert reviewer, affiliation and period of submission of the proposal.
Predicted values from bivariable, unadjusted models (upper panel) and the multivariable analysis (lower panel) are shown, together with their 95% confidence intervals. Scores range from 1 (worst) to 6 (best). Average (mean) overall scores are shown, horizontal lines indicate Wald 95% confidence intervals.

254x190mm (300 x 300 DPI)

Supplementary materials

Table S1: P values from interaction tests of gender of the applicant with other variables, based on bivariable and multivariable models.

	Bivariable	Multivariable*
Gender of reviewer	0.011	0.037
Source of nomination of reviewer	0.71	0.17
Country of affiliation of reviewer	0.57	0.27
Age of applicant	0.74	0.67
Affiliation of the applicant	<0.001	0.003
Nationality of the applicant	0.51	0.92
Research topic	0.36	0.31
Change of guidelines	0.065	0.033

*Adjusted for all variables listed in Table 2 of the main paper.

Table S2: Definition of the field of research.
The field of research is based on the SNSF main discipline list.

Field of research	Main discipline
Medicine	Social medicine, Basic Medical Sciences, Experimental Medicine, Clinical Medicine, Preventive Medicine (Epidemiology/Early Diagnosis/Prevention), Social Medicine
Architecture	Art studies, musicology, theatre and film studies, architecture
Biology	Basic Biological Research, General Biology
Chemistry	Chemistry, Environmental Sciences
Economics	Economics, law
Engineering	Engineering Sciences
Geology	Earth Sciences
History	Theology and religious studies, history, classical studies, archaeology, prehistory and early history
Linguistics	Linguistics and literature, philosophy
Mathematics/Physics	Astronomy, Astrophysics and Space Sciences, Physics, Mathematics, Mathematics, Natural- and Engineering Sciences
Psychology	Psychology, educational studies
Sociology	Sociology, social work, political sciences, media and communication studies, health, Ethnology

For peer review only - http://bmjopen.bmj.com/site/about/guidelines.xhtml

Figure S1: Overall scores depending on research topic and gender of the applicant.

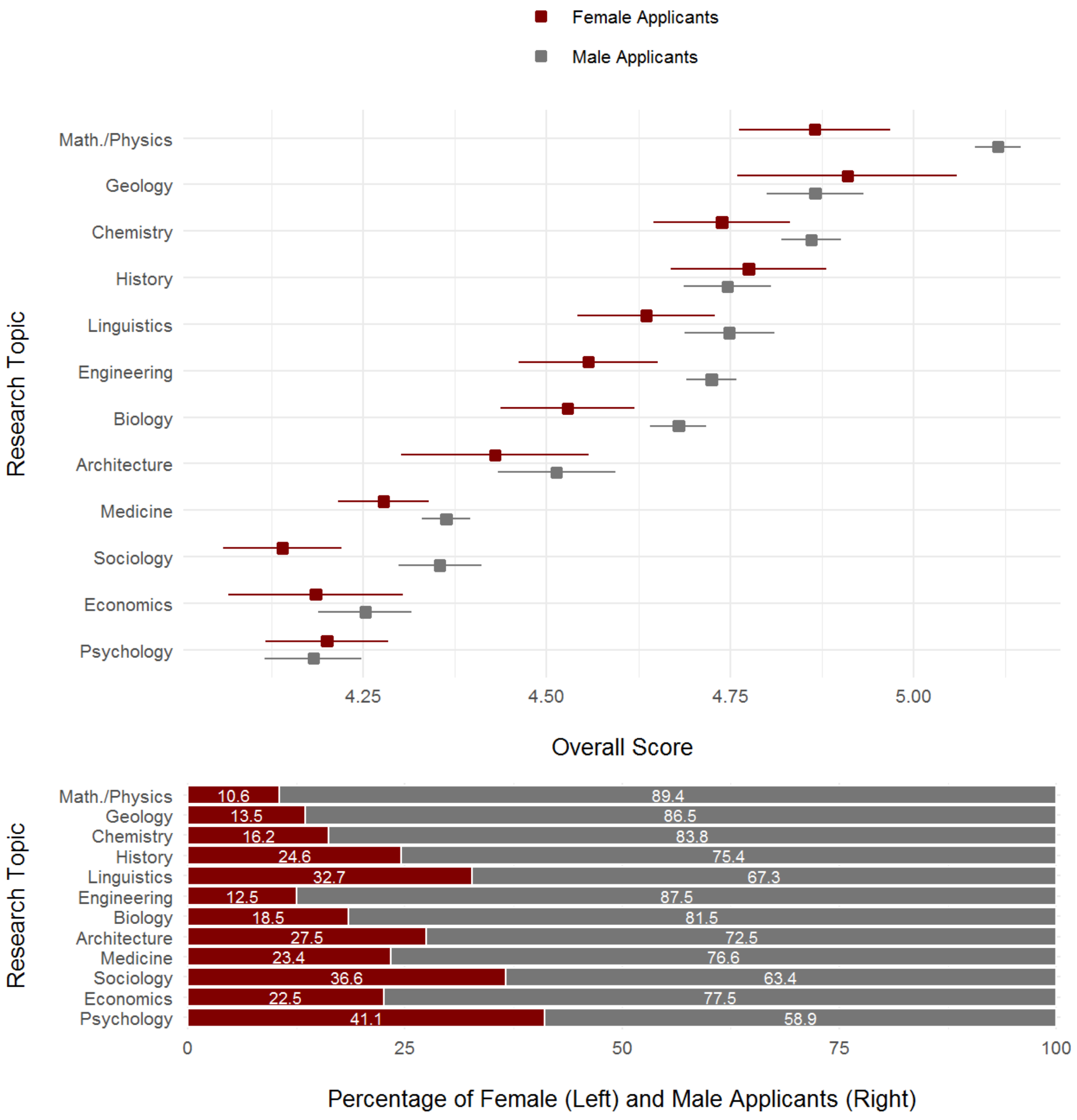

Upper panel: Average (mean) overall scores by research topic for female and male applicants; horizontal lines indicate Wald 95% confidence intervals. Lower panel: Gender distribution in the research topics.

Figure S2: Overall scores depending on the age and gender of the applicants.

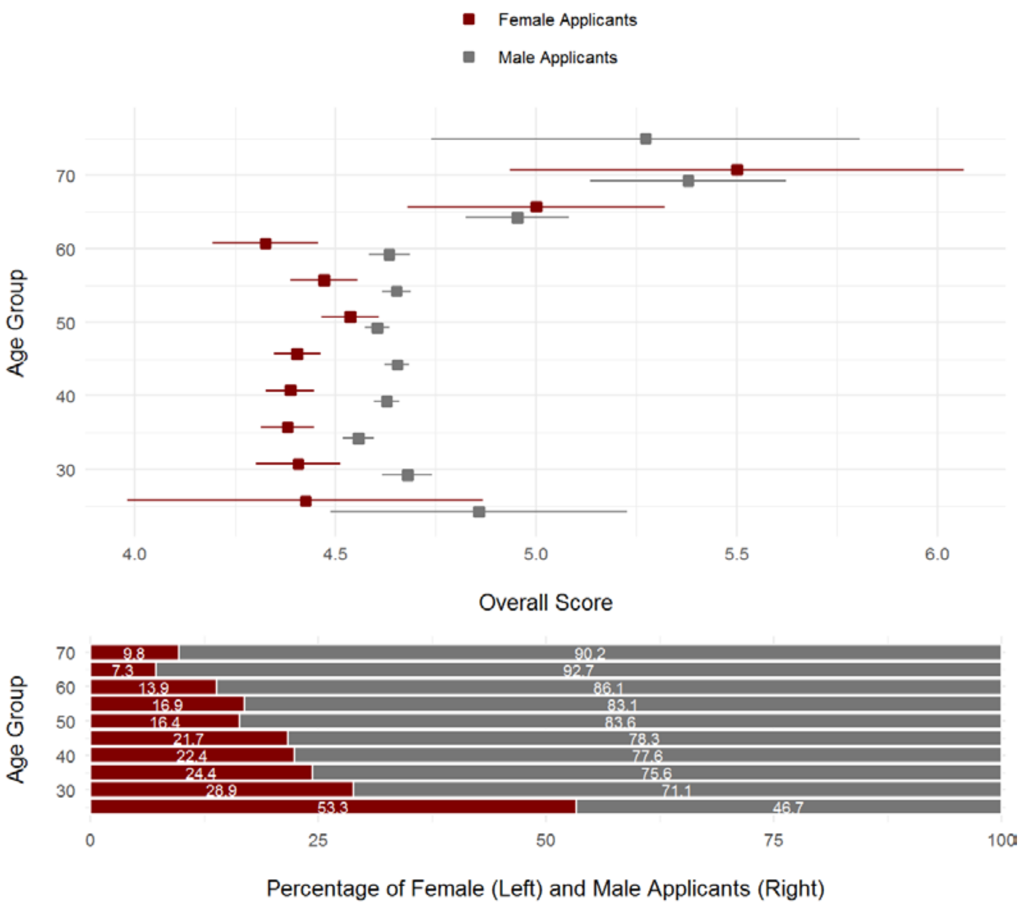

Upper panel: Average (mean) overall scores by five-year age group for female and male applicants; horizontal lines indicate Wald 95% confidence intervals. Lower panel: Gender distribution in the age groups.

For peer review only - http://bmjopen.bmj.com/site/about/guidelines.xhtml

Figure S3: Overall scores depending on the applicant's institution type and gender.

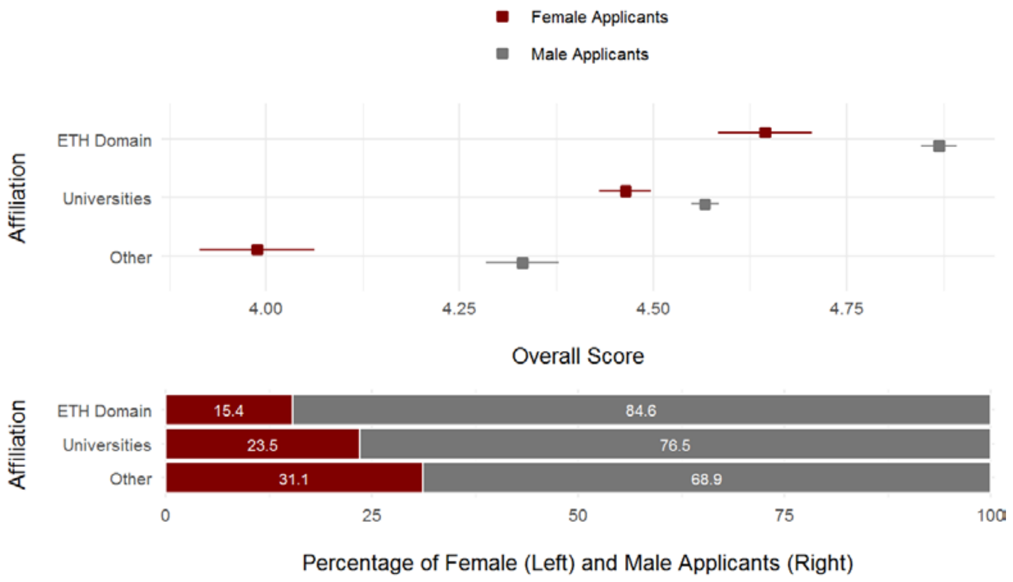

Upper panel: Average (mean) overall scores by affiliation times for female and male applicants; horizontal lines indicate Wald 95% confidence intervals. Lower panel: Gender distribution for the different affiliation types.

For peer review only - http://bmjopen.bmj.com/site/about/guidelines.xhtml

Figure S4: Average overall scores by nationality for female and male applicant; horizontal lines indicate Wald 95% confidence intervals.

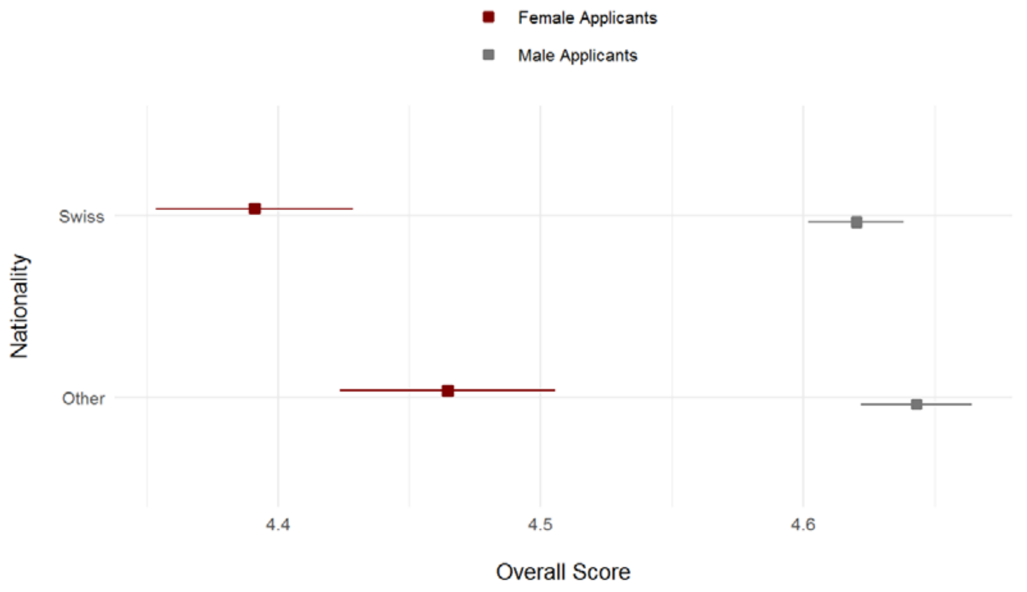

Figure S5: Average (mean) overall scores by application call deadline for female and male applicants; horizontal lines indicate Wald 95% confidence intervals.

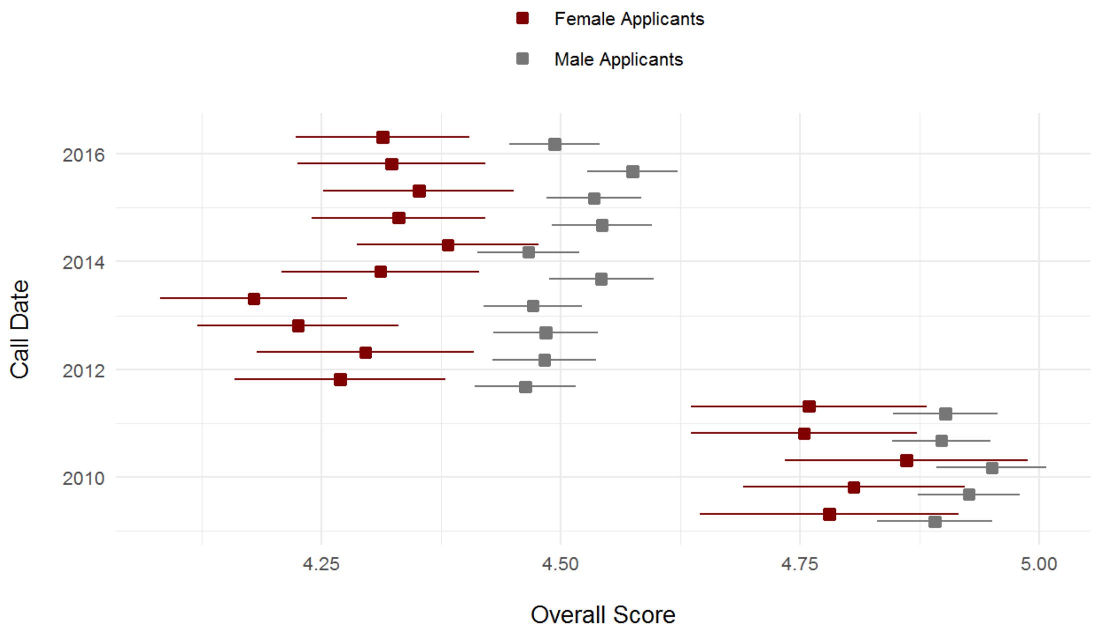

For peer review only - http://bmjopen.bmj.com/site/about/guidelines.xhtml

Text S1: Old and new evaluation forms. The new forms were implemented from the 1. October 2011 call onwards.

OLD FORM

1. Synopsis

	Excellent	Very Good	Good	Average	Satisfactory	Poor		Not considered
Current scientific interest and impact of the project								
Originality of the work								
Suitability and originality of the methods to be used								
Feasibility of the project								
Experience and past performance of the applicant								
Specific abilities of the applicants for the proposed project								

Overall assessment								

For peer review only

Comments regarding the overall assessment

2. Detailed evaluation

Current scientific interest and impact of the project

Originality of the work

Suitability and originality of the methods to be used

Feasibility of the project

Experience and past performance of the applicant

Specific abilities of the applicants for the proposed project

Other comments

For peer review only

For peer review only - http://bmjopen.bmj.com/site/about/guidelines.xhtml

NEW FORM

1. Synopsis

	outstanding	excellent	very good	good	average	poor		Not considered
Applicants' scientific track record and expertise								
Scientific relevance, originality and topicality								
Suitability of methods and feasibility								
Overall assessment								

For peer review only

Comments regarding the overall assessment

2. Detailed evaluation

Applicants' scientific track record and expertise

Scientific relevance, originality and topicality

Suitability of methods and feasibility

3. Further comments & declaration concerning conflicts of interests (will not be forwarded to applicants)

Confidential messages

The topic of the proposed project

Declaration concerning conflict of interests (comments, if applicable

---

For peer review only

STROBE Statement—Checklist of items that should be included in reports of ***cross-sectional studies***

	Item No	Recommendation	Page No
**Title and abstract**	1	(*a*) Indicate the study's design with a commonly used term in the title or the abstract	1
		(*b*) Provide in the abstract an informative and balanced summary of what was done and what was found	2
**Introduction**			
Background/rationale	2	Explain the scientific background and rationale for the investigation being reported	4-5
Objectives	3	State specific objectives, including any prespecified hypotheses	5
**Methods**			
Study design	4	Present key elements of study design early in the paper	6
Setting	5	Describe the setting, locations, and relevant dates, including periods of recruitment, exposure, follow-up, and data collection	5
Participants	6	(*a*) Give the eligibility criteria, and the sources and methods of selection of participants	n.a.
Variables	7	Clearly define all outcomes, exposures, predictors, potential confounders, and effect modifiers. Give diagnostic criteria, if applicable	6
Data sources/ measurement	8*	For each variable of interest, give sources of data and details of methods of assessment (measurement). Describe comparability of assessment methods if there is more than one group	6
Bias	9	Describe any efforts to address potential sources of bias	7
Study size	10	Explain how the study size was arrived at	6
Quantitative variables	11	Explain how quantitative variables were handled in the analyses. If applicable, describe which groupings were chosen and why	7
Statistical methods	12	(*a*) Describe all statistical methods, including those used to control for confounding	7
		(*b*) Describe any methods used to examine subgroups and interactions	7
		(*c*) Explain how missing data were addressed	7
		(*d*) If applicable, describe analytical methods taking account of sampling strategy	n.a.
		(*e*) Describe any sensitivity analyses	n.a.
**Results**			
Participants	13*	(a) Report numbers of individuals at each stage of study—eg numbers potentially eligible, examined for eligibility, confirmed eligible, included in the study, completing follow-up, and analysed	8
		(b) Give reasons for non-participation at each stage	n.a.
		(c) Consider use of a flow diagram	n.a.
Descriptive data	14*	(a) Give characteristics of study participants (eg demographic, clinical, social) and information on exposures and potential confounders	8
		(b) Indicate number of participants with missing data for each variable of interest	8
Outcome data	15*	Report numbers of outcome events or summary measures	8,9, Table 2

For peer review only

Main results	16	(*a*) Give unadjusted estimates and, if applicable, confounder-adjusted estimates and their precision (eg, 95% confidence interval). Make clear which confounders were adjusted for and why they were included	Table 3
		(*b*) Report category boundaries when continuous variables were categorized	n.a.
		(*c*) If relevant, consider translating estimates of relative risk into absolute risk for a meaningful time period	n.a.
Other analyses	17	Report other analyses done—eg analyses of subgroups and interactions, and sensitivity analyses	10
**Discussion**			
Key results	18	Summarise key results with reference to study objectives	11
Limitations	19	Discuss limitations of the study, taking into account sources of potential bias or imprecision. Discuss both direction and magnitude of any potential bias	13
Interpretation	20	Give a cautious overall interpretation of results considering objectives, limitations, multiplicity of analyses, results from similar studies, and other relevant evidence	11-14
Generalisability	21	Discuss the generalisability (external validity) of the study results	13
**Other information**			
Funding	22	Give the source of funding and the role of the funders for the present study and, if applicable, for the original study on which the present article is based	15

*Give information separately for exposed and unexposed groups.

Note: An Explanation and Elaboration article discusses each checklist item and gives methodological background and published examples of transparent reporting. The STROBE checklist is best used in conjunction with this article (freely available on the Web sites of PLoS Medicine at http://www.plosmedicine.org/, Annals of Internal Medicine at http://www.annals.org/, and Epidemiology at http://www.epidem.com/). Information on the STROBE Initiative is available at www.strobe-statement.org.

For peer review only

For peer review only - http://bmjopen.bmj.com/site/about/guidelines.xhtml
